# Ferumoxytol‐Enhanced Myocardial T1 Tracking Using a Hybrid 2D/3D Steady‐State MRI Sequence Captures Cyclic Intramyocardial Blood Volume Dynamics

**DOI:** 10.1002/nbm.70308

**Published:** 2026-05-18

**Authors:** Hazar Benan Unal, Shahriar Zeynali, Abdul Ahmed, Eric Anttila, Ronald Mastouri, Rolf P. Kreutz, Rohan Dharmakumar, David C. Gross, Behzad Sharif

**Affiliations:** ^1^ Laboratory for Translational Imaging of Microcirculation Purdue University Indianapolis Indiana USA; ^2^ Weldon School of Biomedical Engineering Purdue University West Lafayette Indiana USA; ^3^ Division of Cardiovascular Medicine Indiana Univ. School of Medicine Indianapolis Indiana USA; ^4^ MR R&D Collaborations, Siemens Medical Solutions USA Indianapolis Indiana USA; ^5^ Department of Radiology and Imaging Sciences Indiana University School of Medicine Indianapolis Indiana USA; ^6^ MED Institute West Lafayette Indiana USA; ^7^ Bindley Bioscience Center Purdue University West Lafayette Indiana USA; ^8^ Elmore Family School of Electrical & Computer Engineering Purdue University West Lafayette Indiana USA

**Keywords:** blood volume, cardiac cycle, cardiac magnetic resonance, coronary artery disease, ferumoxytol, ischemic heart disease, myocardial MRI, spoiled gradient echo

## Abstract

Measuring cyclic changes in intramyocardial blood volume (iMBV) from systole to diastole has been used as an imaging marker for assessing coronary microcirculation and detecting coronary artery disease (CAD) without the need for vasodilator stress. However, an MRI‐based method for detecting cyclic iMBV dynamics does not exist. The aim of this study is to demonstrate the feasibility of using ferumoxytol‐enhanced (FE) MRI to detect systolic‐to‐diastolic iMBV dynamics on clinical scanners enabled by a new myocardial “T1 tracking” technique. To this end, a continuous steady‐state sequence was developed, combining slice/slab‐selective excitation, to generate high‐resolution T1‐weighted images such that the myocardial signal dynamically tracks the fractional volume of blood while minimizing the influence of confounding factors such as in‐flow effects, through‐plane motion, and spin history. In addition to phantom studies, FE studies in swine (*n* = 10) were conducted to generate systolic/diastolic T1 maps from the T1‐tracking data. For comparison, MOLLI T1 maps were acquired. For both the T1‐tracking method and MOLLI, T1 values before/after ferumoxytol were used to calculate iMBV at end‐systole (ES) and end‐diastole (ED). The T1‐tracking method showed a significant iMBV difference between ES and ED (ES: 6.2 ± 1.8%, ED: 7.7 ± 2.0%, *p* < 10^−3^) as opposed to MOLLI (ES: 8.1 ± 2.9%, ED: 8.6 ± 3.1%, *p* = 0.4), and detected lower iMBV at ES vs. ED in all 10 studies, consistent with physiology, while MOLLI showed contradictory ES‐to‐ED change in 3 out of 10 studies. The proposed method showed a mean iMBV decrease of 19.1% from ED to ES, consistent with the nuclear imaging literature. In conclusion, the results show that the newly developed FE myocardial T1‐tracking technique captures cyclic changes in iMBV, i.e., consistently reveals the expected drop in iMBV from diastole to systole, offering the potential to detect CAD without the need for pharmacological stress.

## Graphical abstract

Cyclic changes in intramyocardial blood volume (iMBV) from systole to diastole can mark coronary disease without the need for vasodilator stress. We present a ferumoxytol‐enhanced MRI technique using a new continuous pulse sequence that effectively tracks cyclic myocardial T1 changes. Preclinical studies on a clinical scanner demonstrate that the proposed technique captures cyclic iMBV changes, consistently revealing the expected drop in iMBV from diastole to systole, whereas conventional MOLLI‐based T1 mapping fails to detect these dynamic variations.

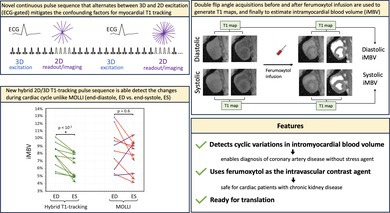

**Abbreviations**
CAD: coronary artery disease
FA: flip angle
FE: ferumoxytol‐enhanced
ES: end‐systole or end‐systolic
ED: end‐diastole or end‐diastolic
iMBV: intramyocardial blood volume
LUT: look‐up table
MOLLI: modified Look‐Locker inversion recovery
PD: proton density
SNR: signal‐to‐noise ratio
SPGR: spoiled gradient recalled echo
SS: steady state

## 1 Introduction

Intramyocardial blood volume (iMBV), defined as the fraction of unit tissue volume occupied by intravascular blood, is an important marker for the status of coronary microcirculation [1, 2]. iMBV connects myocardial blood flow and mean transit time [2, 3] and can be used as a clinical marker to detect various forms of ischemic heart disease including obstructive coronary artery disease (CAD) [4, 5, 6, 7]. The change in iMBV from resting state to the stress state (pharmacological or exercise stress) is referred to as iMBV reserve, which itself can also be used as a biomarker of myocardial ischemia [4, 8, 9]. Under normal physiology, iMBV has a specific temporal dynamic throughout the cardiac cycle: It peaks at end‐diastole (ED), which is when the myocardium is fully relaxed, and reaches its minimum at end‐systole (ES), when the myocardial contraction naturally compresses the intramyocardial microvessels. This cyclic variation is driven by intramyocardial mechanical forces in the ES contraction phase, which reduce iMBV relative to the ED phase [10, 11, 12, 13, 14]. Deviations from this normal pattern indicate abnormal coronary microcirculation which can be due to abnormal vascular tone as a consequence of CAD or due to capillary rarefaction in coronary microvascular disease. Evidence for cyclic change in iMBV from ES to ED has been rigorously observed in preclinical experiments using a variety of non‐MRI techniques and has been proposed as a novel index of coronary microcirculatory function [14, 15, 16, 17, 18]. In fact, prior work in animal models of CAD has shown a direct link between the level of ES‐to‐ED change in iMBV and the severity of disease, i.e., grade of coronary stenosis [19]. Therefore, measuring the cyclic changes in iMBV is a clinically relevant target with the potential to be used as a diagnostic marker for assessing coronary microcirculation and detecting CAD at rest, i.e., without the need for infusion of a vasodilator stress drug [3, 16, 19]. Furthermore, from a basic science perspective, quantifying the relative change in iMBV from systole to diastole has the potential to help improve our understanding of the characteristics of microvascular resistance as well as transmural differences in intramyocardial space [20, 21].

To date, nuclear imaging and contrast‐enhanced ultrasound imaging have dominated the preclinical and clinical approaches for the measurement of iMBV. Specifically, several imaging modalities such as nuclear imaging with radiolabeled “blood pool” tracers, myocardial contrast echocardiography with gas‐filled microbubbles, or volumetric computed tomography are capable of measuring iMBV [2, 22, 23, 24]. Cardiac MRI has the potential to accurately measure iMBV noninvasively without any ionizing radiation when using an intravascular contrast agent such as ultra‐small super‐paramagnetic iron oxide nanoparticles [4]. With the advent of ferumoxytol‐enhanced (FE) MRI, there has been renewed interest in quantifying tissue blood volume fraction, e.g., cerebral blood volume and cerebrovascular reserve [25, 26, 27, 28] as well as iMBV and rest/stress myocardial T1 reactivity, which can serve as a surrogate index of iMBV reserve [4]. In fact, recent preclinical studies [4, 5, 6] have shown the feasibility of using ferumoxytol, which was *approved in October 2025 by the U.S. Food and Drug Administration as the first iron‐based MRI contrast agent*, as a blood‐pool contrast agent for assessment of iMBV by leveraging: (a) its long half‐life, (b) virtually no leakage into the extravascular space unlike gadolinium‐based agents, (c) high signal‐to‐noise ratio (SNR) with steady‐state acquisition.

There is currently no MRI‐based method for detecting the cyclic iMBV dynamics from systole to diastole. The goal of this work is to introduce a clinically translatable myocardial MRI technique for detecting ES‐to‐ED dynamics of iMBV, enabled by a novel continuous pulse sequence and leveraging ferumoxytol as the intravascular agent. Conventional quantitative myocardial MRI pulse sequences, e.g., MOLLI T1 mapping [29], use magnetization preparation across the cardiac cycle which propagates the spin history and may result in inherent insensitivity for accurate detection of cyclic iMBV changes. An alternative approach is to use 2D steady‐state acquisition, e.g., with variable flip angles to track rapid myocardial T1 changes; however, such approaches are confounded by in‐flow effects from rapid dynamics of intra‐ventricular blood and through‐plane motion during systolic contraction. To overcome these confounding factors, the solution that has been previously proposed for brain blood volume studies [30] and cardiac perfusion studies [31, 32] is 3D acquisition since the rapid RF excitation of spins in the entire volume (left ventricle) can keep the spins inside the region of interest (myocardium) at steady‐state magnetization. The downside is that 3D sequences have much higher k‐space sampling requirements vs. 2D acquisition, which implies the need to have undesirably low in‐plane spatial resolution.

Here, we combine the advantages of both 2D and 3D acquisitions by introducing an “instantaneous” T1 mapping technique that is robust to confounding effects of propagation of spin history, inflow and through‐plane motion and can effectively *track myocardial T1 changes* during the cardiac cycle. To this end, we propose a new continuous hybrid 2D/3D pulse sequence *without magnetization preparation* using spoiled gradient‐recalled echo (SPGR) golden‐angle radial readouts to detect cyclic ES‐to‐ED iMBV variations on a clinical scanner enabled by FE imaging. We hypothesize that our proposed approach outperforms MOLLI T1 mapping in myocardial T1 tracking based on the detected ES‐to‐ED iMBV changes. We present quantitative results in large animals *on a clinical scanner*, demonstrating the feasibility of our approach for translational studies.

## 2 Methods

### 2.1 Theory: Estimation of iMBV Under Fast‐Exchange vs. No‐Exchange Conditions

The classic work by Donahue et al. [33] nearly three decades ago used the two‐compartment water exchange model developed by Hazlewood et al. [34] and showed that the fractional volume of the intravascular space inside the tissue can be estimated using pre‐contrast and post‐contrast measurements with an intravascular contrast agent. In the context of myocardial MRI, the tissue, i.e., the myocardium, comprises the intramyocardial microvessels, which is the intravascular space (or compartment), and the extravascular space is the combination of the interstitial space and cardiomyocytes. Here, we apply the framework established by Donahue et al. [33] to FE myocardial imaging.

In this framework, the iMBV is the fractional volume measured using ferumoxytol as the contrast agent. In the limit where there is a *fast water exchange* between the two compartments, the two spin systems act as a single spin system with a uniform T1 [33]. In this setting, the iMBV can be calculated using the following formula:

(1) $$\mathrm{iMB}V_{\text{fast}-\text{exchange}}=\frac{\left(1/T1_{\text{post}-\text{contras}t}^{\text{tissue}}-1/T1_{\mathrm{pre}-\text{contrast}}^{\text{tissue}}\right)}{\left(1/T1_{\text{post}-\text{contrast}}^{\text{blood}}-1/T1_{\mathrm{pre}-\text{contrast}}^{\text{blood}}\right)}=\frac{\Delta \left(1/T1^{\text{tissue}}\right)}{\Delta \left(1/T1^{\text{blood}}\right)}$$

It may be worth pointing out that this T1‐based fast‐exchange formula resembles the myocardial extracellular volume fraction (ECV) formula which uses the pre‐contrast and post‐contrast T1 values using a gadolinium‐based extracellular contrast agent (with an added correction factor to account for the hematocrit) [35] In the opposite limit where there is *no water exchange* between the two compartments, iMBV can be calculated as the ratio of the relative signal‐intensity change from pre‐contrast state to post‐contrast state in tissue vs. blood:

(2) $$\mathrm{iMB}V_{\mathrm{no}-\text{exchange}}=\frac{\left(S_{\text{post}-\text{contrast}}^{\text{tissue}}-S_{\mathrm{pre}-\text{contrast}}^{\text{tissue}}\right)}{\left(S_{\text{post}-\text{contrast}}^{\text{blood}}-S_{\mathrm{pre}-\text{contrast}}^{\text{blood}}\right)}=\frac{\Delta S^{\text{tissue}}}{\Delta S^{\text{blood}}}$$

where S denotes the signal intensity for pre‐contrast and post‐contrast tissue or blood as indicated by subscripts and superscripts.

It should be noted that these two limits (fast exchange in Equation (1) and no exchange in Equation (2)) often do not represent the true in vivo tissue–blood interaction wherein the water molecules travel with a particular exchange rate [5, 34, 36]. As a result, iMBV can be overestimated or underestimated depending on the assumption used for the exchange regime. One approach to overcome this challenge is to achieve the so‐called “exchange independence” condition which, for SPGR sequences, has been derived analytically as follows [33]:

(3) $$\frac{\mathrm{TR}}{T1}\;\frac{\cos\left(\alpha \right)}{\left(1-\cos\left(\alpha \right)\right)}\ll 1$$

where T1 is intravascular longitudinal relaxation time, α is the flip angle (FA), and TR is the repetition time for the SPGR pulse sequence. When the exchange independence condition is satisfied, the two iMBV values derived using the fast‐exchange assumption as in Equation (1) and the no‐exchange assumption as in Equation (2) will be identical. For a given T1 and TR, one indication that we have approximately satisfied this condition is to observe that $\mathrm{iMB}V_{\text{fast}-\text{exchange}}$ and $\mathrm{iMB}V_{\mathrm{no}-\text{exchange}}$ have nearly converged to the same value. We will use this theoretical insight specifically in the two‐compartment phantom studies described below (to identify the ground‐truth fractional volume in a mechanical phantom) and to demonstrate that our proposed SPGR pulse sequence is able to quantify the ratio of two fractional volumes accurately.

### 2.2 Proposed Sequence for Myocardial “T1 Tracking”: Continuous Spoiled Steady‐State With Hybrid 2D/3D Excitation

Fast SPGR pulse sequences with 2D slice‐selective acquisition are commonly used in myocardial MRI; however, their T1‐weighted signal is confounded by through‐plane motion and in‐flow effects, where the out‐of‐slice unsaturated spins result in signal variation throughout the cardiac cycle. Our goal is to develop a pulse sequence capable of generating high‐resolution T1‐weighted images such that the myocardial signal intensity dynamically tracks the fractional volume of blood, which means we need to minimize the out‐of‐slice unsaturated spins to eliminate these confounding factors. To this end, and inspired by prior work on steady‐state myocardial perfusion MRI with continuous acquisition [32, 37, 38], we developed a “hybrid” sequence consisting of slice‐exciting (2D) and slab‐exciting (3D) RF pulses with continuous tiny golden‐angle (≈ 23.63°) radial acquisition [39]. We used SPGR readout instead of balanced steady‐state free precession readout (despite the SNR penalty) to avoid any banding/susceptibility artifacts in presence of ferumoxytol at 3 T.

**FIGURE 1:**
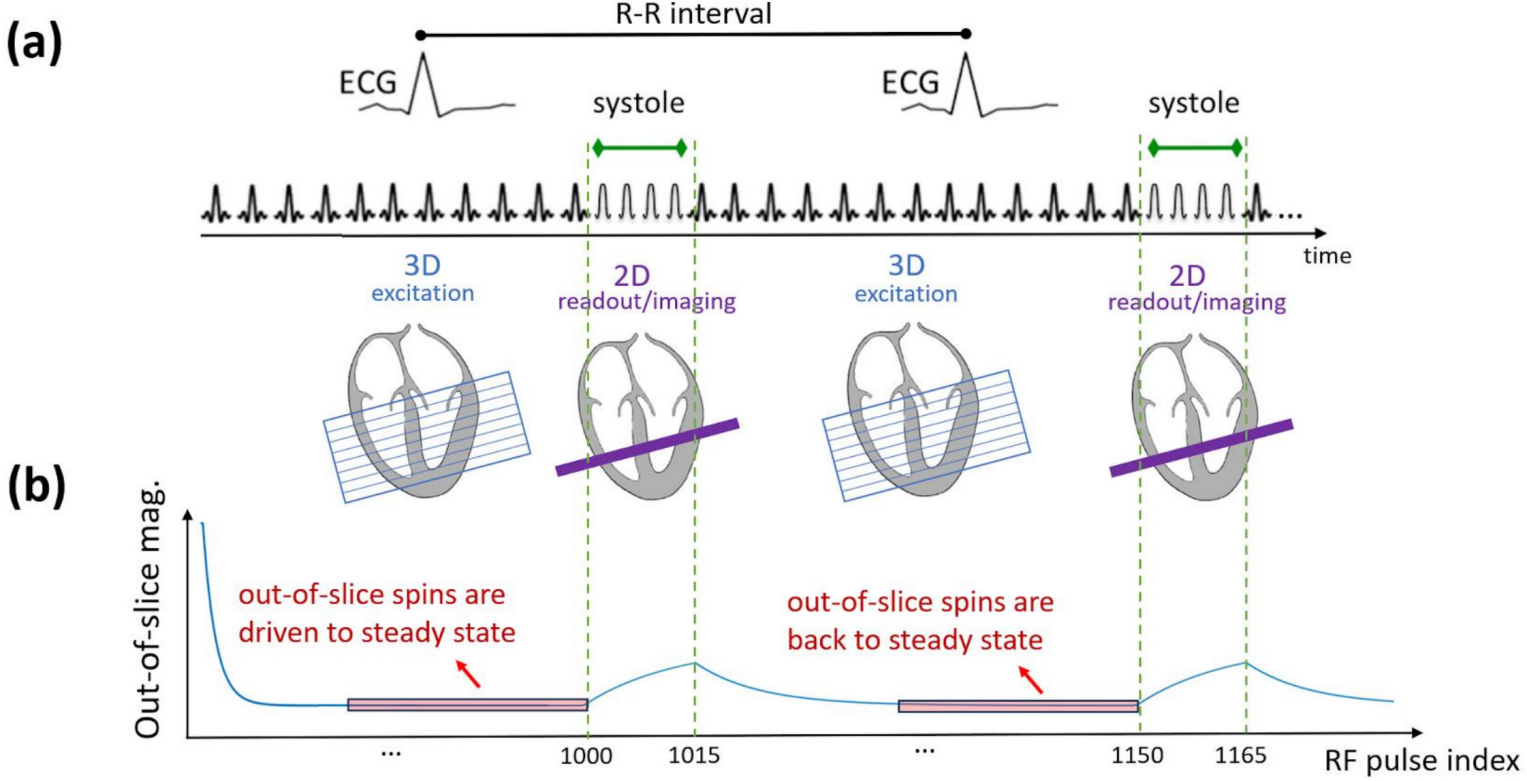
Conceptual description of the proposed continuous steady‐state pulse sequence for myocardial “T1 tracking” with hybrid 2D/3D excitation, and how it periodically maintains the out‐of‐slice magnetization in steady‐state prior to end‐systolic readout/imaging. (a) End‐systolic hybrid 2D/3D pulse sequence wherein 3D and 2D RF pulses are applied continuously with golden‐angle radial RF‐spoiled gradient‐recalled echo (SPGR) readouts. The proposed sequence starts with a series of rapid 3D excitation pulses to drive all spins in the volume (covering the whole heart, shown as a blue stack) to steady‐state, then switches between 2D and 3D pulses based on a user‐defined “2D readout/imaging” interval (to image the 2D short‐axis slice, shown in purple) that is synchronized with the R‐wave to enable systolic (as shown) or diastolic (not shown) imaging. (b) The magnetization state in the spins outside of the 2D imaging slice is schematically described; a key feature of the proposed hybrid 2D/3D sequence is that the 3D excitation pulses ensure that the out‐of‐slice magnetization (which is disrupted during 2D excitation) returns to steady state before the next 2D‐imaging interval. This in turn ensures robustness to confounding effects of blood in‐flow and through‐plane motion.

Figure 1 provides a conceptual description of the new continuous spoiled steady‐state pulse sequence for myocardial “T1 tracking” and how it periodically maintains the out‐of‐slice magnetization in steady‐state using 3D excitation, prior to a brief ES readout/imaging using 2D slice‐selective excitation. As demonstrated in Figure 1, the 3D pulses in the proposed pulse sequence ensure that the out‐of‐slice magnetization (which is disrupted when switching to 2D excitation during the brief 2D readout/imaging period) returns to steady state before the next 2D readout/imaging interval (in the next heartbeat). The 3D pulses are only used to drive the out‐of‐slice spins to steady‐state and are not used in the image reconstruction process.

**FIGURE 2:**
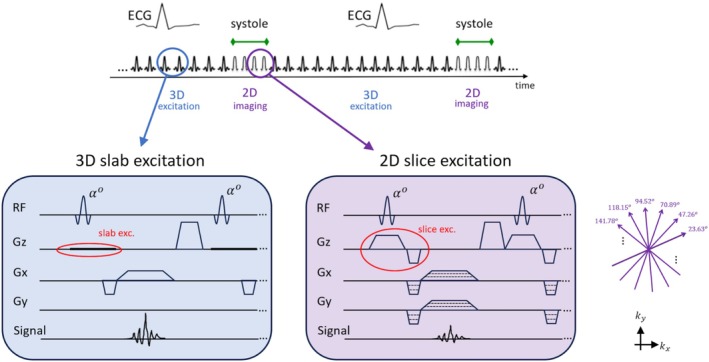
Methodology for the proposed continuous spoiled steady‐state pulse sequence for myocardial T1 tracking without magnetization preparation and hybrid 3D (slab selective) and 2D (slice selective) RF excitation. The proposed pulse sequence consists of hybrid of slab‐exciting (3D) and slice‐exciting (2D) SPGR sequences. RF pulses with the same flip angle are applied continuously (i.e., no magnetization preparation) with a tiny golden‐angle radial trajectory (23.63° angular increments), where steady‐state RF‐spoiled 3D excitation switches to 2D excitation only for a short “slice readout” period that is adjustable by scanner user interface. To perform end‐systolic imaging, for example, the time interval between the R‐wave and the end‐systolic phase is estimated using a 2D cine scout scan and is entered in the user interface for the proposed pulse sequence to place the 2D slice readout period at the end‐systolic phase as shown here. A similar approach enables end‐diastolic imaging (not shown) synchronized with the R‐wave. Continuous 3D pulses keep the out‐of‐slice spins in steady state, and 2D readouts are used for image reconstruction for single slice.

Figure 2 shows the details of the 3D (slab selective) and 2D (slice selective) RF excitation blocks for the proposed myocardial T1 tracking pulse sequence, and describes how the proposed sequence switches between 2D and 3D RF excitation pulses based on a user‐defined “2D imaging” interval synchronized with the R‐wave to enable ES imaging. Both Figure 1 and Figure 2 show the methodology for ES imaging; the method for ED imaging is similar with a delayed “trigger delay” relative to the R‐wave, i.e., with the 2D readout/imaging period adjusted to be in the ED phase. The pulse sequence was implemented on a clinical 3 T scanner (MAGNETOM Verio, Siemens Healthineers, Erlangen, Germany) with TR/TE set to be 4.5/1.7 ms (with small deviation for in vivo studies) and adjustable flip angle. At the reconstruction stage, the acquired 2D radial k‐space lines with golden‐angle increments from all heart beats are combined (for ES and ED images separately) and reconstructed using a re‐gridding kernel and conjugate‐gradient non‐Cartesian SENSE [40]. Additional implementation details include the following: (1) The trigger delay was programmed to be an adjustable parameter in the pulse‐sequence user interface to enable both ES and ED imaging; (2) in the first 5 s of the scan, continuous 3D pulses run (no 2D pulses) as an “initialization” to ensure that all spins in the slab are driven to steady‐state magnetization; (3) a temporal footprint < 100 ms for ES/ED imaging was achieved as an additional design criterion, which limited the maximum number of 2D readouts per heartbeat to 22 projections. A typical in vivo evolution of the total magnetization using the implemented pulse sequence (acquired signal intensity from a 3 T pig study) is provided in Supporting Information under Suppl. Figure S1, which shows the 3D excitation pulses indeed drive the total signal intensity back to steady‐state before 2D imaging starts in the next heartbeat.

### 2.3 Proposed Approach: T1 Quantification and iMBV Estimation

To generate the myocardial T1 map for the ES phase, we acquire data using the proposed pulse sequence twice: once with proton‐density (PD) weighting (very low FA, selected to be 3°) and once with T1‐weighting (high FA, selected to be 20°). In the next step, we generate T1 maps by using a similar approach as the one performed in SR‐prepared SPGR acquisition for fully quantitative myocardial perfusion MRI [41]: We generate a look‐up table (LUT) based on Bloch equation simulations for the SPGR sequence to map the PD‐normalized myocardial signal intensities to a T1 value. As is the case for gadolinium‐based myocardial perfusion imaging [42], normalization by the PD‐weighted image effectively eliminates T2*‐weighting and coil‐weighting effects as part of the T1 fitting process. To estimate iMBV at the ES phase, data acquired using the proposed pulse sequence before ferumoxytol infusion (referred to as the native or “pre‐FE” scan) and after ferumoxytol infusion (referred to as the “post‐FE” scan) are combined using the fast‐exchange formulation as described above in Equation (1) [33]. This process is then repeated for the ED phase data acquisition to estimate iMBV at the ED phase.

### 2.4 Simulations

We performed Bloch equation simulations to (a) find the highest feasible heart rate for different FAs to ensure that the out‐of‐slice spins (which are excited by the continuous 3D pulses) reach steady‐state (with a tolerance of 5%) before 2D readout/imaging starts in each R‐R interval, and (b) investigate the myocardial “T1 tracking” ability (i.e., how accurately the changes in T1 during cardiac cycle can be detected) of different flip angles for different heart rates. Consistent with the design for the proposed hybrid 2D/3D sequence, we set TR/TE = 4.5/1.7 ms and used 22 projections per heartbeat in the simulations (temporal footprint: 99 ms). To model the dynamically changing iMBV throughout the cardiac cycle—specifically, higher iMBV during diastole vs. systole—we simulated periodically changing T1 values that vary within ±10% of a fixed mean value (post‐FE myocardial T1 of 1000 ms). We then performed the proposed LUT‐based T1 tracking approach using Bloch equation simulations with realistic RF and gradient characteristics for two different FAs (low and high) for the periodically changing signal. We ran the simulations for both low and high heart rates (50 bpm and 100 bpm).

To investigate the effect of fast‐exchange assumption on relative ES‐to‐ED iMBV change under in vivo water exchange conditions, we performed simulations using the two‐compartment exchange model developed by Hazlewood et al. [34] and later refined by Bjornerud et al. [43]. We adopted the average in vivo water exchange rate in porcine myocardium which was estimated by Colbert et al. [5] using ferumoxytol‐enhanced CMR on similar 3 T hardware with the same animal species and similar body weight range. The other parameters such as blood T1 or iMBV were selected based on the results of our in vivo experiments. Finally, to assess the robustness of the proposed method to slice profile imperfections [44, 45], we performed Bloch equation simulations with realistic sinc‐shaped RF pulses with varying time‐bandwidth products and evaluated the sensitivity of the estimated fractional volume (simulated iMBV) to imperfections in slice profile.

### 2.5 Two‐Compartment Phantom Studies

**FIGURE 3:**
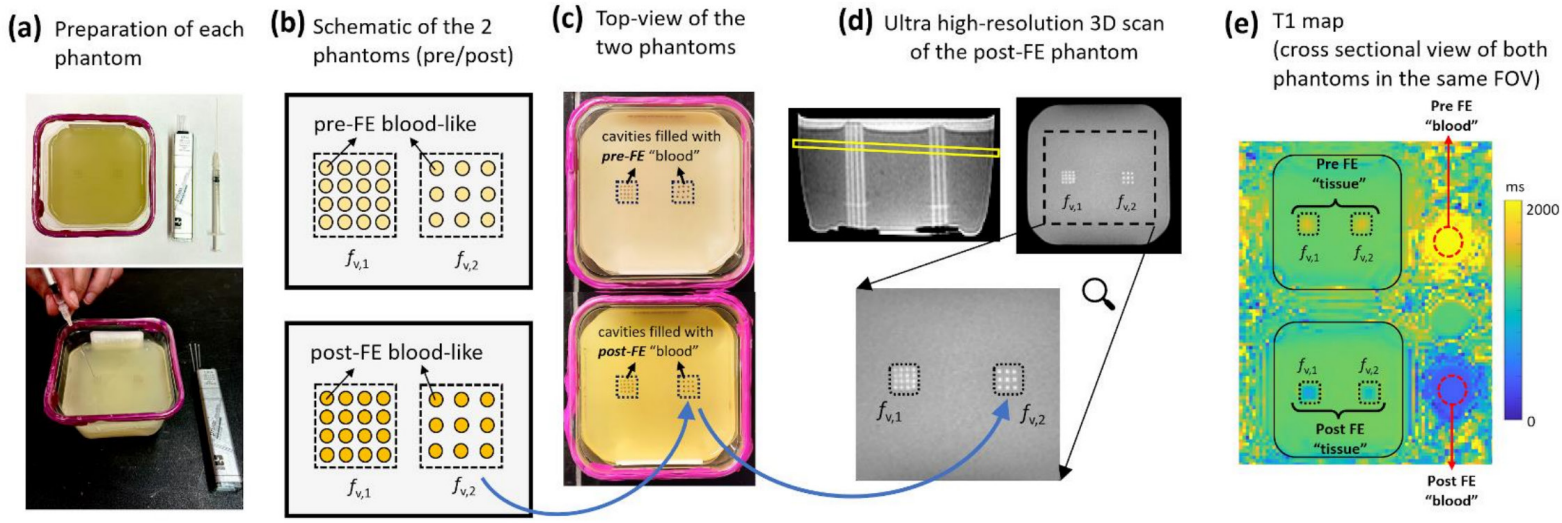
Description of phantom experiments to investigate the impact of water exchange regime on the measured “iMBV ratio” using a realistic fractional volume model. (a) We built a physical phantom using gadolinium‐doped agar and ferumoxytol‐doped saline to model extravascular space and intravascular space, respectively, as the two compartments that constitute the myocardium. To mimic the capillaries inside the myocardium filled with and without the intravascular contrast agent, we used capillary tubes (glass cylinders in the silver box) to create cylindrical cavities (holes) in agar gel. (b) We designed the pre‐contrast (pre‐FE) phantom at the top and the post‐contrast (post‐FE) phantom at the bottom, with two different fractional volume values (*f*_v,1_ and *f*_v,2_) where the circles represent the cross‐sections of the intravascular space inside the myocardium. (c) Top‐view of the two agar‐based phantoms (the post‐FE phantom at the bottom), each with the two sets of cavities, creating two sets of fractional volume quantities; the top phantom was submerged in a pre‐contrast (pre‐FE) blood‐like solution and the bottom phantom in a post‐contrast (post‐FE) blood‐like solution so that the intravascular space in both phantoms were filled with pre/post contrast blood‐like solution. (d) Ultra‐high‐resolution 3D scan for post‐FE phantom verifies that the cavities filled with post‐FE blood‐like solution have shorter T1 resulting in a brighter signal; the bottom picture is the zoomed‐in view of the 2D cross‐section (highlighted in yellow in the side view) of the post‐FE phantom showing the same pattern of intravascular cross‐sections as intended in the design step in panel (b). (e) After confirming that the cylindrical cavities were filled without any air bubbles, we acquired a low‐resolution T1 map so that two compartments (intravascular vs. extravascular) are not spatially resolved, mimicking the in vivo setting for myocardial T1 mapping.

The proposed method for detecting the ES‐to‐ED iMBV ratio relies on the fast‐exchange assumption. We conducted elaborately designed phantom studies to investigate the impact of water‐exchange regime (i.e., deviation from the fast‐exchange assumption) on the accuracy of iMBV ratio measurements using the proposed approach. Figure 3 describes how we built a physical phantom with a realistic fractional volume model using contrast‐doped agar/saline and cylindrical cavities to model extravascular and intravascular spaces. Description of the phantom design and experiments are provided in Supporting Information under Appendix A.

### 2.6 In Vivo Studies

In vivo MRI studies were performed using anesthetized domestic swine (*n* = 10; weight: 38 ± 7 kg) on a whole‐body 3 T clinical scanner (MAGNETOM Verio, Siemens Healthineers, Erlangen, Germany) with a standard cardiac‐torso receiver coil array. All imaging experiments were performed under a protocol approved by the local Institutional Animal Care and Use Committee. Prior to MRI scans, all animals were fasted, sedated, intubated, anesthetized, and positioned on the scanner table in a feet‐first position. Throughout the MRI scans, the animals were mechanically ventilated with a mixture of oxygen and isoflurane (1.5%–2%) to maintain anesthesia. Continuous monitoring of heart rate, blood pressure, oxygen saturation, and temperature was performed using a physiological monitoring unit (InVivo Expression MR, Philips, Cambridge, Massachusetts, United States) throughout the imaging session. The mean heart rate across all 10 pig MRI studies was 101 ± 7 beats per minute.

We used ferumoxytol (Feraheme, Azurity Pharmaceuticals, Woburn, Massachusetts, United States) as T1‐shortening contrast agent to create two different contrast regimes (pre and post) needed for iMBV mapping. After localization of a single mid‐ventricular short‐axis slice, we ran a 2D cine scan to obtain the time‐points for ES and ED phases, followed by a B1+ calibration scan [46]. Data was acquired using the proposed hybrid 2D/3D pulse sequence at ES and ED phases (PD‐weighted with FA = 3° and T1‐weighted scan with FA = 20°) before ferumoxytol infusion with high spatial and temporal resolution: (a) in‐plane spatial resolution of 1.4 × 1.4 mm^2^ (32% improvement from typical 1.7 × 1.7 mm^2^ with MOLLI); (b) a temporal footprint of ≈ 80 ms by acquiring 18 projections per heartbeat. We then administered 1:10 diluted ferumoxytol (≈ 2 mg/kg) slowly over 15 min. Following a 20–25‐min waiting period, we acquired data using the same sequence, separately at ES and ED phases. The measurement time for each individual scan varied between 20 and 30 s, depending on the heart rate. Next, ES and ED iMBV maps were generated using the above‐described “T1 quantification and iMBV estimation” section. For comparison, we also acquired standard breath‐hold MOLLI T1 maps at ES and ED phases. For per‐vessel analysis, the results were analyzed based on three regions of interest (ROIs) corresponding to myocardial territories supplied by each of the three epicardial coronary arteries. For per‐subject analysis, a single ROI was used covering the LV myocardium. All the image reconstruction and analysis were performed in MATLAB (MathWorks, Natick, Massachusetts, United States). The results were reported as mean ± standard deviation. Student's *t*‐test was performed for group comparisons, and a *p* value smaller than 0.05 was considered statistically significant. The reproducibility of the proposed approach was investigated on three animals by retrospectively truncating the acquired raw k‐space data (projections) into three consecutive scan segments and measuring the coefficient of variation for the relative ES‐to‐ED iMBV change across the three sequential segments of the scan in the same animal.

## 3 Results

### 3.1 Simulations

**FIGURE 4:**
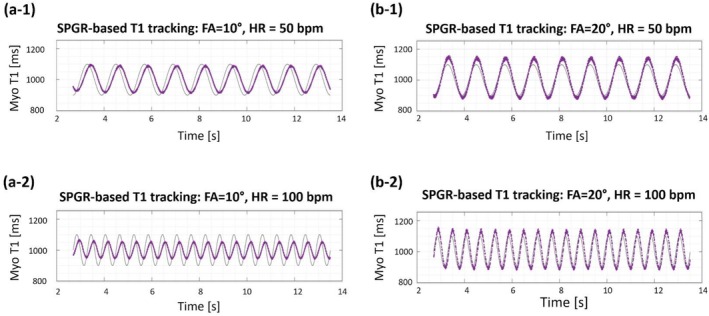
Simulation results for optimizing the choice of flip angle (FA) in the proposed SPGR‐based myocardial T1 tracking approach. To investigate the performance of the proposed SPGR‐based approach for tracking the periodic changes in myocardial T1 due to cyclic iMBV dynamics (lower iMBV in ES phase and higher iMBV in ED phase), we performed a series of simulations with different FAs (10° and 20°) and heart rates (50 bpm and 100 bpm). The “ground truth” myocardial T1 was set to be a sinusoidal function with a given frequency (determined by the heart rate), a mean value of 1000 ms for post‐FE myocardium, and a dynamic range of ±10% deviation from mean throughout the cardiac cycle. We then applied the proposed LUT‐based T1 mapping technique using Bloch equation simulations with realistic RF and gradient characteristics to quantify myocardial T1 at each time point, i.e., to perform “instantaneous” myocardial T1 mapping (solid curve for ground‐truth T1 and purple curve for estimated T1). In panels (a‐1) and (a‐2), which use a lower FA of 10°, the speed of T1 tracking is slower which results in underestimation of peak/trough in the cyclic T1 dynamics when the heart rate is high (lower panel). In contrast, in panels (b‐1) and (b‐2), which use a higher FA of 20°, the speed of T1 tracking is sufficient to capture the rapid T1 dynamics even at the high heart rate of 100 bpm.

The first set of simulations, which investigated the range of heart rates that allow the out‐of‐slice spins to reach steady‐state before 2D readout/imaging starts, showed that the highest feasible heart rate was 183 bpm for FA = 20° and 103 bpm for FA = 3°. The results of the second set simulations are summarized in Figure 4. Briefly, the numerical experiments showed that using low FA = 10° results in slower tracking of the periodically changing T1 (solid curves as ground truth in Figure 4), which leads to underestimation of peak/trough in the cyclic T1 dynamic when the heart rate is high (Figure 4[a‐1,a‐2]). Using a relatively high FA of 20°, on the other hand, achieves a near “instantaneous” T1 tracking with minimal delay as shown in Figure 4(b‐1,b‐2) for the high heart rate of 100 bpm. These simulations confirm the choice of FA = 20° for the T1‐weighted scans in the in vivo experiments as a solid compromise between T1‐tracking ability and SNR.

The simulations with a two‐compartment model comparing the relative iMBV change in the fast‐exchange and in vivo water exchange conditions are presented in Supporting Information (Suppl. Figure S2). Results of the simulation study evaluating the sensitivity of the estimated fractional volumes to imperfections in the slice profile are presented in Supporting Information (Suppl. Figure S3).

### 3.2 Phantom Studies

**FIGURE 5:**
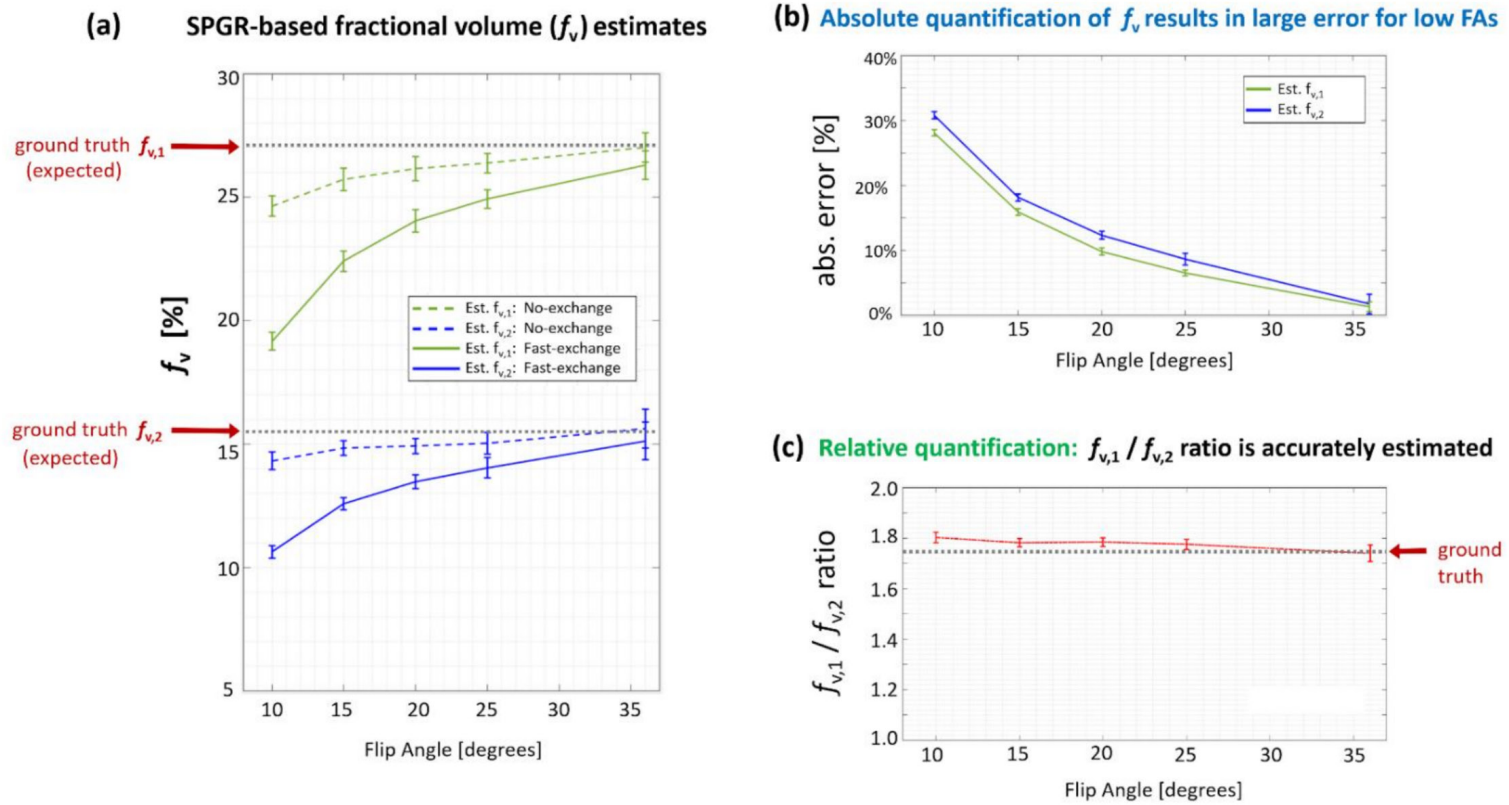
Results of the phantom experiments comparing the quantification accuracy of the proposed SPGR‐based technique to estimate absolute fractional volume versus ratio of two fractional volumes. (a) Estimated fractional volumes in the two regions‐of‐interest denoted by *f*_v,1_ and *f*_v,2_ in Figure 3d (left and right square‐shaped regions) as a function of the flip angle (FA) derived from the phantom experiments, demonstrating a monotonic behavior as expected, with the maximum FA dictated by SAR limitation. Consistent with the physics of water exchange [33], higher FAs provide exchange‐independence in SPGR‐based fractional volume estimation since iMBV values estimated using the closed‐form expressions for the two extreme regimes (no‐exchange shown with dashed lines vs. fast‐exchange shown with solid lines) converge to the ground truth fractional volume as the FA is increased, i.e., where the dashed and solid curves meet. This observation provides us with the knowledge of the expected ground truth values for *f*_v,1_ and *f*_v,2_, which are used in panels (b) and (c). (b) Plot of the estimation error for the two fractional volumes, *f*_v,1_ and *f*_v,2_, as FA in the SPGR sequence increases; as can be seen, the FA has a major impact on the accuracy of the estimated absolute fractional volume (underestimation by up to 30% at the low FA of 10°). (c) Plot of the ratio of the two fractional volumes, i.e., *f*_v,1_ divided by *f*_v,2_, as FA in the FA in the SPGR sequence increases; in contrast to (b), the ratio is quantified accurately (1.78 vs. 1.73, i.e., < 3% error) and is relatively independent of the FA (within 5%). This in turn implies that the proposed SPGR pulse sequence (with the choice of FA = 20°) is capable of accurately quantifying the ratio of two fractional volumes, supporting the reliability of our ES‐to‐ED relative iMBV quantification in the subsequent in vivo studies.

**FIGURE 6:**
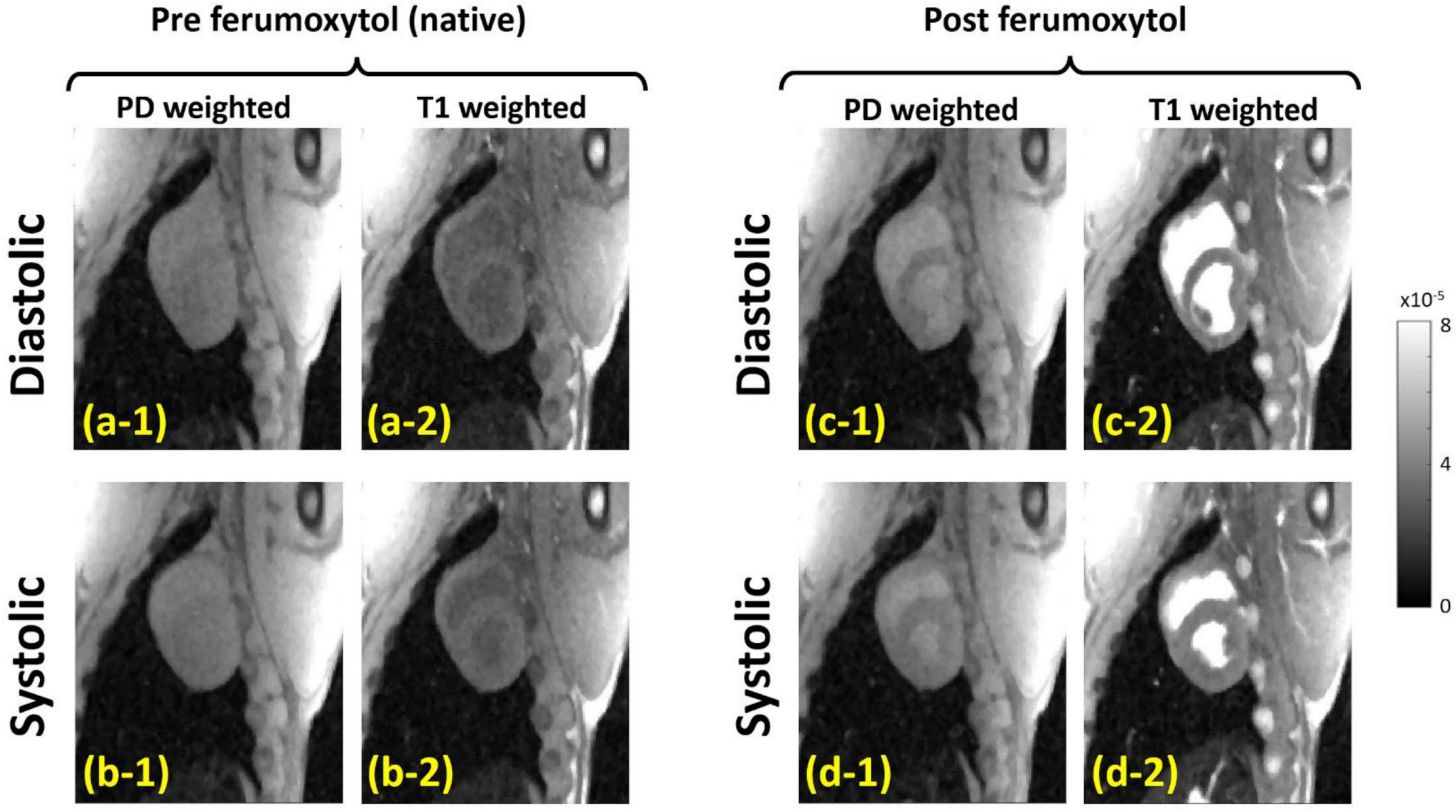
Representative in vivo case showing reconstructed end‐systolic (ES) and end‐diastolic (ED) images acquired before and after ferumoxytol infusion (pre‐FE and post‐FE) using the proposed hybrid 2D/3D myocardial T1‐tracking pulse sequence. We acquired proton‐density (PD) weighted and T1‐weighted images in diastolic and systolic phases both at (a,b) pre‐FE and (c,d) post‐FE. The “dark blood” appearance observed in the T1‐weighted pre‐FE images (a‐2) and (b‐2) reflects effective suppression of in‐flow and through‐plane motion effects, attributable to the 3D excitation pulse train incorporated in the proposed sequence. Note the consistent ES/ED phase across the four acquired images in each row. The high quality of the reconstructed images can be attributed to the brief 2D readout/imaging period (minimal cardiac motion blur) and the golden‐angle radial trajectory (enables efficient k‐space oversampling in the angular direction).

In the two‐compartment phantom studies using the realistic fractional volume design (Figure 3), we first obtained the expected ground‐truth fractional volumes *f*_v,1_ and *f*_v,2_ for the two square‐shaped ROIs described in Figure 3d based on the theoretical derivations in Methods. Specifically, we applied the understanding that the estimated fractional volumes using the fast/no exchange assumptions converge to the ground‐truth fractional volume value as FA is increased, i.e., as we enter the exchange‐independent regime (Equation (3)). As shown in Figure 5a, the fractional volumes estimated using the fast‐exchange (dashed lines) vs. no‐exchange (solid lines) formulas (Equation (1) and Equation (2), respectively) for both *f*_v,1_ and *f*_v,2_ (green and blue colors) start to converge to the same value as FA is increased, which is consistent with the theoretical framework. The maximum FA here is dictated by the SAR limitations on the scanner. For *f*_v,1_ and *f*_v,2_ separately, we extrapolated each curve to a theoretical (infeasible) FA = 90° and took the mid‐point between the two curves to be the expected ground‐truth fractional volume. This resulted in 26.9% and 15.5% for *f*_v,1_ and *f*_v,2_, respectively, resulting in a ground‐truth fractional volume ratio of 1.73. Next, we examined how the estimation error varied as function of FA for the *absolute* quantification of two fractional volumes vs. *relative* quantification of fractional volume ratio. The results shown in Figure 6b,c demonstrate that the error in absolute fractional volume estimation could reach up to 30% for low flip angles ≈ 10° (Figure 5b). However, as shown in Figure 5c, the relative quantification of fractional volume ratio is quantified accurately for FA = 20° (1.78 vs. 1.73, i.e., < 3% error) and is relatively consistent across all FAs (within 5%). This finding indicates that, while the proposed T1‐tracking method may not yield *accurate* absolute iMBV values, it can accurately detect the *relative* iMBV change between ES and ED phases. This phenomenon can be explained by the simple observation that a *ratio of two quantities* is unchanged if both the numerator and denominator are underestimated or overestimated by similar relative amounts. Yet, another observation from the phantom studies is that the standard deviation of the measurements increased at higher FAs (Figure 5a). This trend is expected since the Ernst angle for pre‐FE and post‐FE T1 values in the SPGR sequence is relatively low (between 5° and 10°). Combined with the SAR limitations on the whole‐body clinical 3 T scanner, the SNR penalty associated with higher FAs further confirmed using moderately high FAs (i.e., FA = 20°) for T1‐weighted images for the in vivo studies.

### 3.3 In Vivo Studies

Figure 6 shows representative images for ES and ED cardiac phases before and after ferumoxytol infusion. PD‐weighted and T1‐weighted images were obtained during diastolic (top row) and systolic (bottom row) phases before ferumoxytol (a,b) and after ferumoxytol infusion (c,d). The “dark blood” effect seen in the pre‐FE T1‐weighted images indicates successful suppression of in‐flow and through‐plane motion artifacts, enabled by the 3D excitation pulse train used in the proposed imaging sequence. Notably, each row shows consistent ES and ED phases across the four images. The high image quality is enabled by the short 2D readout/imaging window, which minimizes cardiac motion blur, combined with the golden‐angle radial trajectory that allows for effective k‐space oversampling in the angular dimension.

**FIGURE 7:**
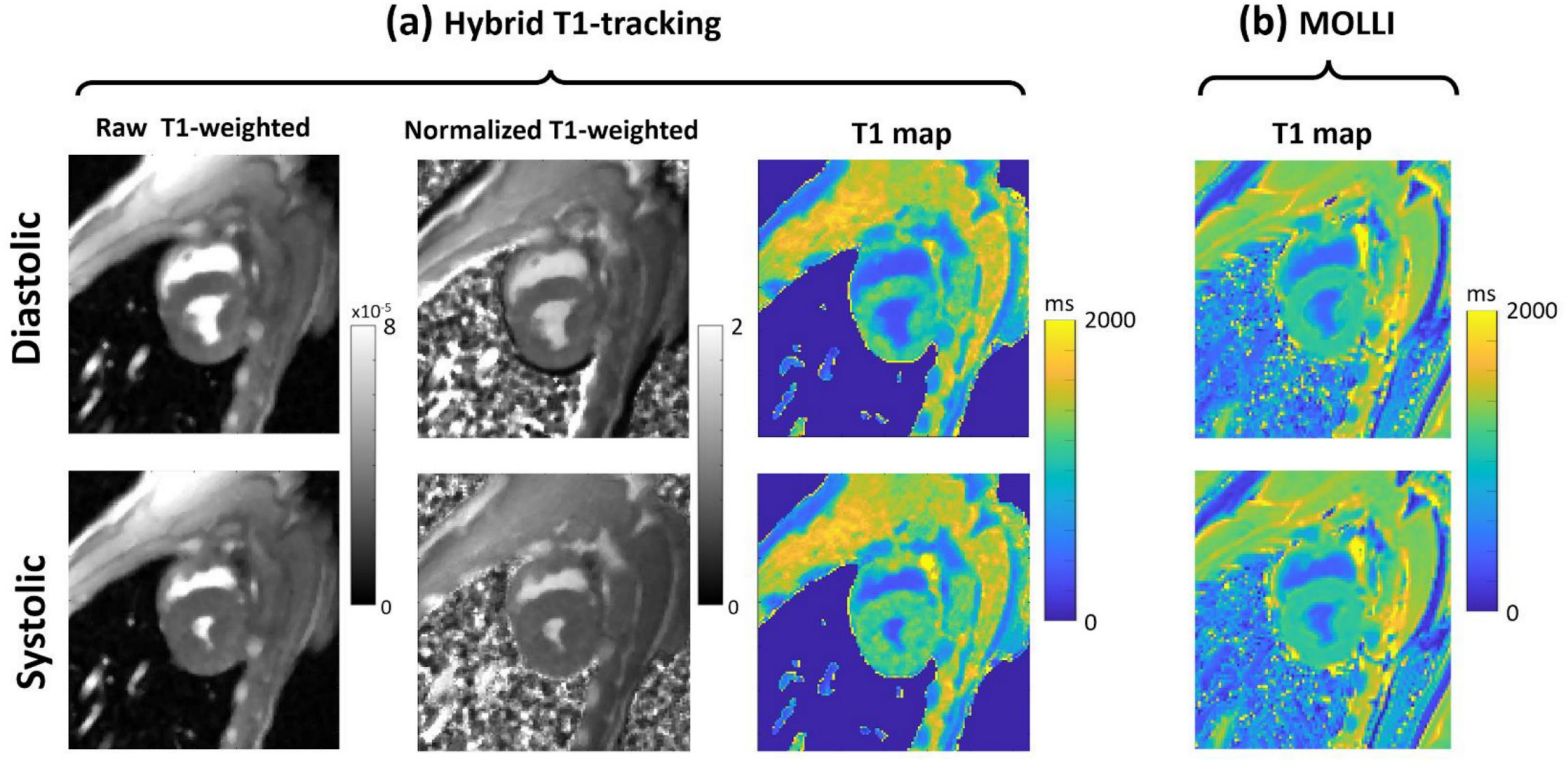
Example systolic and diastolic T1 maps generated using the proposed hybrid 2D/3D myocardial T1‐tracking approach with comparison to standard MOLLI for a second representative case. (a) The raw and “normalized” T1‐weighted image (i.e., T1‐weighted image divided by PD‐weighted image) are shown; the normalized T1‐weighted image is mapped to T1 values using a look‐up table based on Bloch equation simulations. (b) MOLLI myocardial T1 maps acquired with two different trigger delays to obtain systolic and diastolic T1 maps. Compared to MOLLI, the T1 maps generated using proposed hybrid 2D/3D method have sharper edges along the endocardial border, which can be attributed to the shorter data acquisition window (the 2D readout/imaging period in the proposed method) during each R‐R interval (82 ms for the proposed method vs. 135 ms for MOLLI). Furthermore, unlike MOLLI, the systolic/diastolic T1 maps generated by the proposed myocardial T1‐tracking method are robust to the confounding effects of propagation of spin history, in‐flow and through‐plane motion—all of which help detect the cyclic changes in iMBV.

Figure 7 shows example T1 maps (systolic and diastolic) obtained using the proposed hybrid 2D/3D myocardial T1‐tracking approach and standard MOLLI for another representative case (post‐FE). Figure 7a shows the raw T1‐weighted and “normalized” T1‐weighted image for the proposed T1‐tracking method, which are then mapped to T1 values using LUT‐based Bloch equation simulations. Figure 7b shows the myocardial MOLLI T1 maps acquired with two different triggers delays for systolic and diastolic imaging. Compared to MOLLI, the proposed T1‐tracking approach provides sharper edges along the endocardial border. This can be attributed to the shorter data acquisition window in the proposed T1‐tracking approach during each R‐R interval (82 ms vs. 135 ms for MOLLI). Moreover, the systolic and diastolic T1 maps acquired using the proposed T1‐tracking method are robust to the confounding effects of propagation of spin history, in‐flow and through‐plane motion, which help detecting the cyclic changes in iMBV.

**FIGURE 8:**
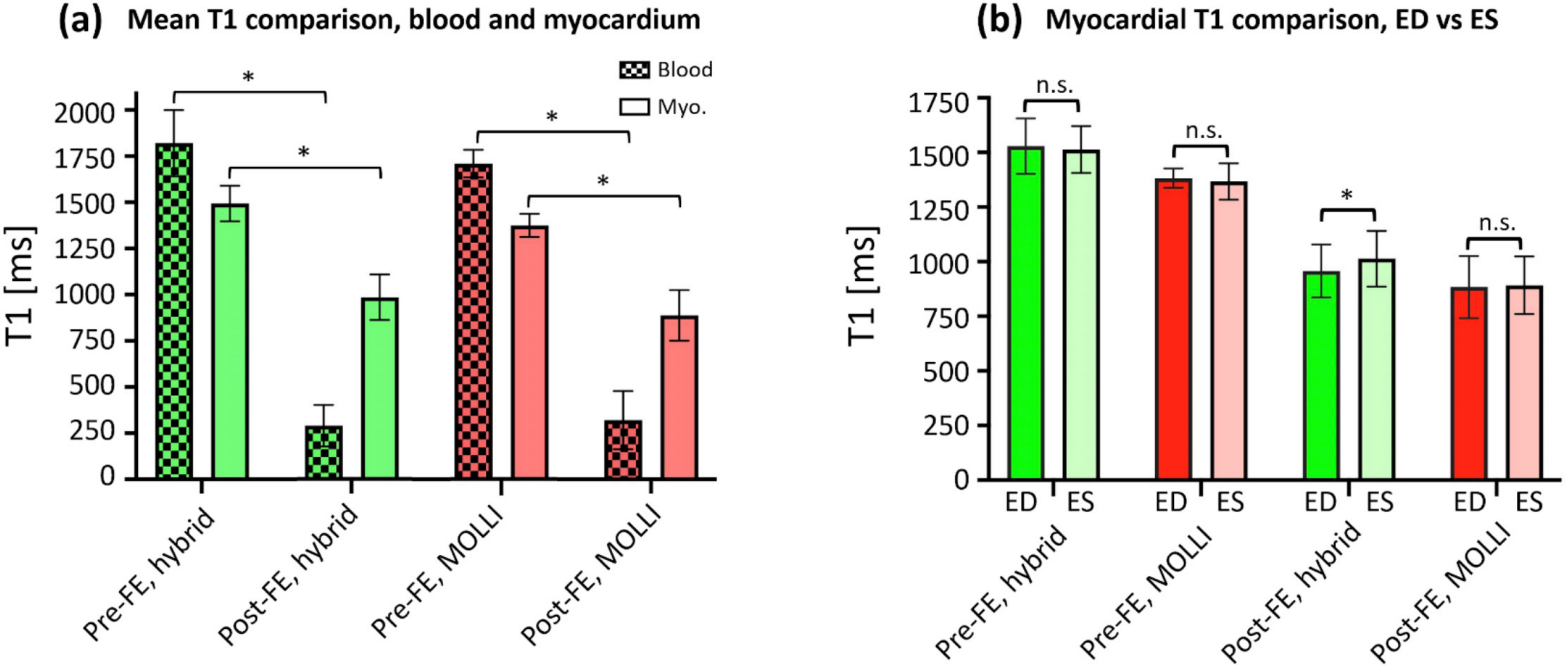
Comparison of measured T1 values in the in vivo studies (*n* = 10 pigs) using the proposed T1‐tracking hybrid 2D/3D sequence and standard MOLLI. (a) Pre‐FE and post‐FE T1 values for blood and myocardium measured using the proposed T1‐tracking hybrid 2D/3D sequence (green) and MOLLI (red) are shown. As expected, ferumoxytol significantly shortens blood T1 (checkerboard bars) and myocardial T1 (solid‐colored bars) with *p* < 10^−4^ for all comparisons. (b) Comparison of diastolic vs. systolic myocardial T1 values for the proposed T1‐tracking hybrid 2D/3D sequence and MOLLI before and after ferumoxytol infusion (pre‐FE and post‐FE scans). Among the four paired comparisons, only the post‐FE myocardial T1 measured using the proposed myocardial T1‐tracking method resulted in a significant difference (*p* < 0.01) between end‐diastole (ED) and end‐systole (ES) phases, which demonstrates the feasibility of detecting ES‐to‐ED iMBV dynamics using the proposed approach.

Figure 8 shows the comparison of measured T1 values in the in vivo studies using the proposed T1‐tracking method and MOLLI. Figure 8a shows the measured blood and myocardial T1 values (ES and ED combined) using proposed T1‐tracking method and MOLLI at pre‐FE and post‐FE scans. In the pre‐contrast state, the proposed T1‐tracking method estimated blood T1 = 1820 ± 179 ms and myocardial T1 = 1493 ± 97 ms; MOLLI estimated blood T1 = 1709 ± 74 ms and myocardial T1 = 1374 ± 64 ms. In the post‐FE scans, proposed T1‐tracking method estimated blood T1 = 289 ± 111 ms and myocardial T1 = 985 ± 123 ms; MOLLI had blood T1 = 320 ± 157 ms and myocardial T1 = 888 ± 136 ms. As expected, both proposed T1‐tracking method and MOLLI showed significantly shorter blood and myocardial T1 values at post‐FE scans vs. pre‐FE scans (*p* < 10^−4^). Figure 8b shows the systolic and diastolic myocardial T1 values using proposed T1‐tracking method and MOLLI before and after ferumoxytol infusion. In the pre‐FE scans, proposed hybrid 2D/3D sequence had myocardial T1 = 1513 ± 99 ms in ES phase, myocardial T1 = 1529 ± 117 ms in ED phase (*p* = 0.45); and MOLLI had myocardial T1 = 1368 ± 77 ms in ES phase, myocardial T1 = 1383 ± 41 ms in ED phase (*p* = 0.38). In the post‐FE scans, proposed hybrid 2D/3D sequence had myocardial T1 = 1014 ± 117 ms at ES phase and T1 = 957 ± 112 ms at ED phase (*p* < 0.01); MOLLI had myocardial T1 = 892 ± 122 ms at ES phase and myocardial T1 = 884 ± 131 ms at ED phase (*p* = 0.37). Notably, among the four paired comparisons, only post‐FE myocardial T1 values measured with the proposed hybrid 2D/3D T1‐tracking method was able to distinguish the myocardial T1 differences at ES vs. ED cardiac phases. This demonstrates the feasibility of detecting ES‐to‐ED iMBV changes using the proposed approach.

**FIGURE 9:**
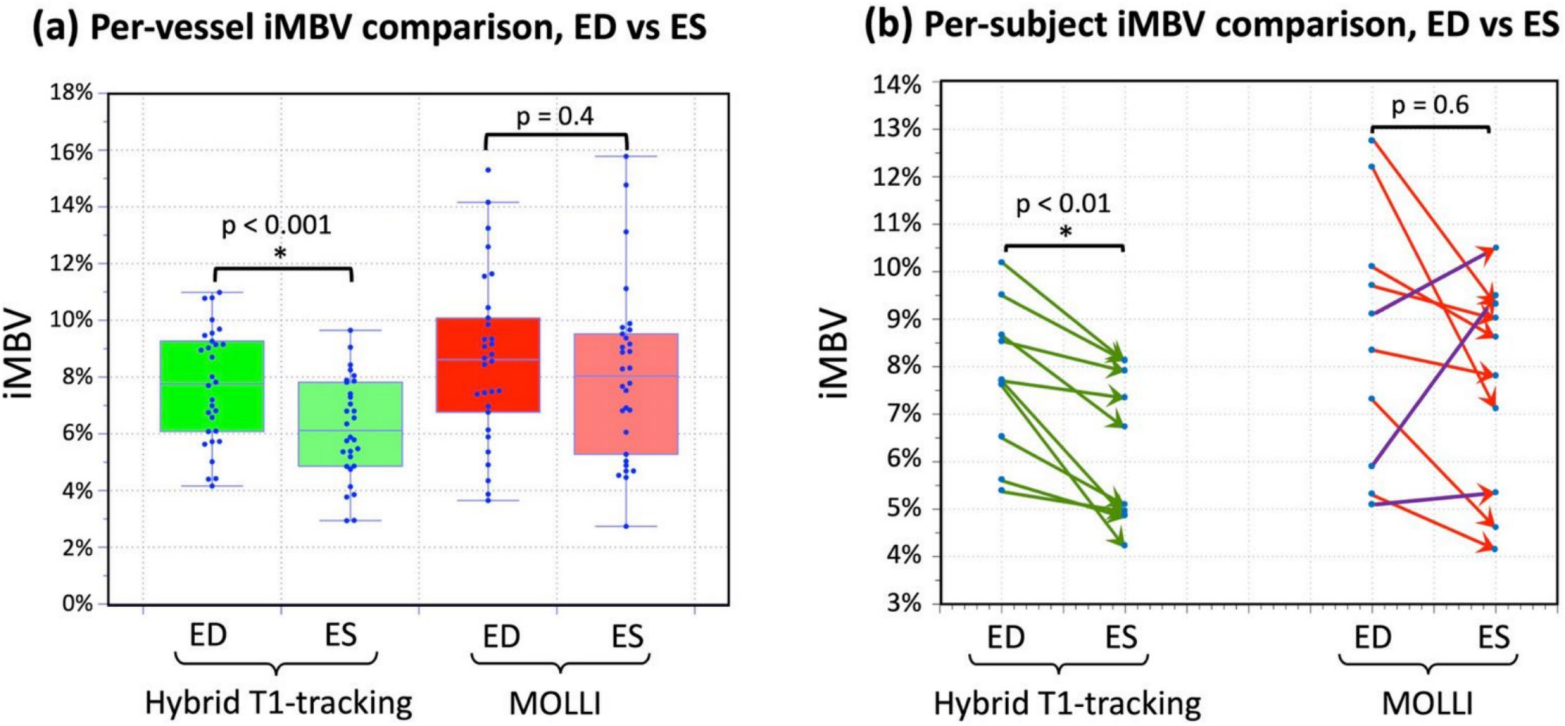
Summary of the in vivo results to evaluate the feasibility detecting cyclic changes in iMBV from the ES phase to the ED phase. (a) For per‐vessel analysis, a total of 30 iMBV measurements are reported (3 coronary artery territories, 10 studies) as shown by blue dots in the boxplot with scatter plot overlay. The iMBV comparison between ED and ES acquisitions for the proposed hybrid 2D/3D T1‐tracking approach are shown on the left side with green boxes (ED = 7.7% ± 2.0%, ES = 6.2% ± 1.8%, *p* < 10^−3^) and the measurements for MOLLI are shown on the right side with red boxes (ED = 8.6% ± 3.1%, ES = 8.1% ± 2.9%, *p* = 0.4). As can be seen, the proposed method is able to detect the ED‐to‐ES iMBV drop (19.1% relative change, p < 10^−3^) whereas MOLLI does not show a significant drop (*p* = 0.4). (b) For per‐subject analysis, the average iMBV change across all three coronary territories from ED to ES is shown for both methods. As can be seen, the proposed method detected a significant drop in iMBV from ED to ES in all 10 subjects (*p* < 10^−2^) as expected from the physiology. MOLLI, however, did not show a significant difference between ES and ED (*p* = 0.6). Moreover, in 3 out of 10 studies, MOLLI resulted in contradictory cyclic iMBV changes, i.e., demonstrating a measured ES iMBV that was higher than the measured ED iMBV.

Figure 9 describes the summary of the in vivo experiments to evaluate the feasibility of detecting cyclic changes in iMBV from ES phase to ED phase. As shown in Figure 9a, in the per‐vessel analysis, the proposed myocardial T1‐tracking method resulted in a significant difference between iMBV at ES vs. ED (ED = 7.7% ± 2.0%, ES = 6.2% ± 1.8%, *p* < 10^−3^) as shown with green boxes. On the other hand, MOLLI did not show a significant difference between ES and ED phases (ED = 8.6% ± 3.1%, ES = 8.1% ± 2.9%, *p* = 0.4) as shown with red boxes. As shown in Figure 9b, in the per‐subject analysis, the proposed T1‐tracking method consistently showed ES iMBV to be lower than ED iMBV (as expected from physiology) for all 10 studies. On average, there was 19.1% ± 12.5% significant change in iMBV from ED to ES with proposed T1‐tracking method (*p* < 10^−2^). In contrast, for MOLLI, the ES iMBV was higher than ED iMBV for 3 of the 10 studies, which indicates that MOLLI‐derived iMBV does not reflect the cyclic iMBV dynamics accurately. Although iMBV decreased from ED to ES by 14.5% ± 36.7% on average with MOLLI, the difference was not statistically significant (*p* = 0.6). The results of the repeatability experiments for the proposed method are provided in Supporting Information (Suppl. Figure S4).

## 4 Discussion

The presented results in this work indicate that the proposed myocardial T1‐tracking method, using a continuous steady‐state pulse sequence with hybrid 2D/3D excitation, can detect periodic changes in iMBV throughout the cardiac cycle with FE imaging. This is enabled by a spoiled steady‐state T1 contrast mechanism using rapid 3D excitations without magnetization preparation, combined with brief 2D readouts at a specific cardiac phase, so that the myocardial signal intensity dynamically tracks the fractional volume of blood while minimizing the influence of confounding factors which, in turn, enables our approach to quantify iMBV at end‐systole or end‐diastole. The presented large animal studies on a clinical scanner, using ferumoxytol as the intravascular contrast agent, show an average of 19.1% decrease in iMBV from ED to ES (Figure 9a) which is consistent with a recent nuclear imaging study in large animals [15] (reported 23.9%) and within the range reported in the prior literature in animals and humans [47, 48]. In contrast, as shown in Figure 9b, MOLLI‐derived iMBV did not show a significant difference between ES and ED and, in 30% of the studies, resulted in contradictory ES/ED iMBV changes, i.e., showing a measured ES iMBV that was larger than the measured ED iMBV. Based on this observation, our results indicate that the proposed approach outperforms MOLLI T1 mapping in tracking the myocardial T1 changes from ES to ED in ferumoxytol‐enhanced CMR. This can be attributed to the fact that, unlike the proposed myocardial T1‐tracking method which uses a steady‐state contrast mechanism (rapid SPGR) that can capture “instantaneous” T1 maps (as described in simulation results in Figure 4), the magnetization preparation pulses in MOLLI effectively mix the T1‐weighting between ES and ED phases, i.e., they propagate the spin history from ES to ED and vice versa (described graphically in Suppl. Figure S5). The inability of MOLLI to detect a significant difference between myocardial T1 at ES vs. ED is consistent with similar studies in the literature [49, 50, 51]. In fact, previous theoretical work demonstrated that high flip‐angle SPGR sequences are more effective at capturing instantaneous changes in T1 than inversion‐recovery sequences [52].

Prior preclinical and clinical studies using contrast‐enhanced ultrasound techniques have established a direct link between the level of ES‐to‐ED change in resting iMBV and the severity of coronary artery disease, i.e., grade of coronary stenosis [19, 53]. Therefore, detection of cyclic changes in iMBV is a clinically significant target with the potential to be used as a diagnostic marker for ischemic heart disease without administration of a stress agent [3, 16, 19, 53]. Furthermore, detecting the relative change in iMBV from ES to ED has the potential to help improve our understanding of the characteristics of microvascular resistance as well as transmural differences in the intramyocardial vascular network [20, 21, 54]. Recent work using nuclear modalities has shown the feasibility of detecting the cyclic dynamics of iMBV variation, from its trough at ES to its peak at ED, in humans with potential clinical applications [15, 48]. In this work, the presented results provide a solid proof‐of‐concept to support the feasibility of quantifying cyclic iMBV changes on clinical MR scanners. The proposed approach is ready for translation in human studies, which can be performed without breath‐holding since the myocardial tissue remains within the excited 3D volume during shallow free‐breathing scans (the imaged 2D slice will be in steady state). Supplementary Figure S6 provides preliminary in vivo evidence on the feasibility of a free‐breathing extension of the proposed method. In our preclinical studies, we acquired the ES and ED images in separate scans due to the elevated resting heart rate in pigs (101 ± 7 bpm). Given the longer resting R‐R interval in human subjects, in clinical studies this data can be acquired in the same scan (i.e., two 2D image readouts per R‐R interval, one at ES and one at ED) with optimized pulse sequence parameters.

Previous work using established CMR techniques has provided indirect evidence suggesting cyclic iMBV changes, e.g., by showing differences in myocardial perfusion or native myocardial T1 between ES vs. ED phases [49, 55]. Furthermore, ultra‐high field CMR at 7 T has shown ES vs. ED differences in myocardial T2* values [56]. It should be noted that T2*‐based approaches can be confounded by other factors including field inhomogeneity. Further, such changes in T2* may primarily reflect the difference in the BOLD effect rather than iMBV changes. The direct investigation of cyclic iMBV dynamics under CMR, however, has so far been limited to small animal studies using non‐clinical scanners and investigational contrast agents that are not commercially available [47]. Our proposed methodology, for the first time, enables the detection of ES/ED iMBV dynamics on a clinical 3 T scanner with promising results, demonstrating ES vs. ED differences in the expected physiologic range. Furthermore, our approach uses ferumoxytol as an intravascular contrast agent, which is being increasingly adopted in clinical studies. These two features imply that the proposed technique is well positioned for translation to clinical settings.

The mean blood and myocardial T1 values measured in the presented in vivo studies are in the expected range based on: (a) the MOLLI‐derived T1 values in the same animals; (b) prior studies in large animals at 3 T. As can be seen from Figure 9a, the pre‐FE myocardial T1 values measured using the hybrid 2D/3D method are higher than the MOLLI‐measured pre‐FE myocardial T1. This is due to the lack of the confounding effect of magnetization transfer in the hybrid 2D/3D method (which uses an SPGR readout), which in the case of MOLLI, has been shown to result in reduced pre‐FE T1 [57]. An important result that can be observed from mean T1 plots is the relatively high variability of pre‐FE blood T1 using the proposed T1‐tracking method. This is due to lower SNR at the pre‐FE acquisition, especially for the SPGR readout used in the proposed T1‐tracking method. However, the high variability in pre‐FE blood T1 does not affect the estimated iMBV using the fast‐exchange assumption significantly thanks to the ferumoxytol dose we administered. In fact, even a 200‐ms underestimation of a true pre‐FE blood T1 of 1800 ms only affects the resulting fast‐exchange iMBV estimate by less than 3% (relative error) if post‐FE blood T1 is lower than 350 ms, which was the case in nearly all of the in vivo experiments.

Coronary circulation primarily occurs during the diastolic phase. In our results, we observed noticeable variation in iMBV quantified using the proposed method across different coronary territories in the ED phase; specifically, for the proposed method, the iMBV for the myocardial territory supplied by the left anterior descending (LAD) coronary artery is approximately 19% higher than the territory supplied by the left circumflex (LCX) coronary artery (LAD: 8.3% ± 1.8% vs. LCX: 7.09% ± 1.8%, *p* < 0.05). This observation is consistent with a recent study in pigs using FE CMR which aimed to accurately quantify absolute iMBV using multi‐compartment modeling [5], and is also in line with the expected variation of coronary circulation in swine [58].

### 4.1 Limitations

A potential limiting factor for the accuracy of iMBV estimated using the proposed method is the assumption of fast water exchange between the intravascular and extravascular compartments, which can result in over/underestimation of the absolute iMBV values [5, 33]. However, it should be noted that the focus of this study was to investigate the relative iMBV change from the ES phase to the ED phase rather than performing accurate absolute iMBV quantification at a specific cardiac phase. In fact, the result of our realistic phantom experiments (mimicking the intramyocardial micro‐vessels) indicate that the relative ratio between two iMBV values (e.g., ES iMBV relative to ED iMBV) is mostly insensitive to the exchange regime assumption (unlike absolute iMBV values). Additionally, simulation results (Suppl. Figure S2) based on a well‐established exchange model showed that there is less than 5% relative difference (1% absolute difference) between the in vivo exchange regime vs. fast exchange regime when it comes to measuring the relative ES‐to‐ED iMBV change. Limitations for RF energy deposition (SAR) can potentially affect the proposed method especially at 3 T, which is why the “high flip angle” acquisition in our study was limited to 20°. Since SAR has a quadratic relationship with the magnetic field strength, mid‐field or low‐field CMR can accommodate much higher flip angles and improve the accuracy for absolute iMBV quantification provided that the penalty on SNR is somehow compensated (e.g., using advanced denoising algorithms, higher ferumoxytol dose, etc.). This is because a large enough flip angle may satisfy the exchange‐independence condition described in Equation (3), hence providing a more reliable exchange‐independent estimation of absolute iMBV values as reflected in our phantom results in Figure 5a. Since the proposed method needs a correction for the flip angle during signal‐to‐T1 conversion, the errors due to B1+ inhomogeneities can be mitigated by using more advanced B1+ correction techniques [59] or a hardware approach, e.g., using parallel transmit or using low‐field/mid‐field MRI scanner platforms (e.g., 0.55 T) to reduce B1+ inhomogeneities [60, 61]. We used a fixed ferumoxytol dose of 2 mg/kg in our pig imaging studies since this dose was previously shown to have the highest correlation between fast‐exchange estimated iMBV vs. in vivo iMBV in pigs on ferumoxytol‐enhanced CMR [5]. Although the dose–response relationship was not investigated in this study, the range of 2–4 mg/kg is becoming increasingly more common in ferumoxytol‐enhanced MRI studies at high‐field strengths, with an excellent safety profile in line with gadolinium agents [62, 63, 64, 65]. Moreover, there is in vivo evidence that mid‐field MRI can potentially enable lower ferumoxytol doses [66]. Finally, this study lacks comparison to a gold‐standard method, and future work may explore the comparison between CMR and other imaging modalities.

## 5 Conclusion

In this work, we proposed a ferumoxytol‐enhanced myocardial MRI framework employing a new hybrid 2D/3D steady‐state SPGR pulse sequence with golden‐angle radial acquisition to detect cyclic intramyocardial blood volume (iMBV) dynamics, specifically the change in iMBV from systole to diastole. Our results demonstrate that the proposed methodology, for the first time, enables the detection of ES‐to‐ED iMBV changes on a clinical scanner. The clinical value of quantifying ES‐to‐ED iMBV changes is known based on prior work derived from non‐MRI modalities. This, combined with the fact that ferumoxytol is being increasingly adopted in clinical MRI studies, points to the translational potential of the proposed methodology in clinical settings involving patients with suspected coronary artery disease or microvascular dysfunction.

## Author Contributions

Initial concept and theoretical derivations (HBU, BS); development and implementation of the MRI pulse sequences for data acquisition (HBU, BS); phantom studies (HBU, SZ, AA, EA, DCG, BS); preclinical imaging studies (HBU, SZ, EA, RM, RPK, RD, DCG, BS); interpretation of data and results (HBU, AA, RM, RPK, RD, BS); drafting of the manuscript (HBU, BS); critical reviewing of the manuscript (all authors); Approval of the submitted version (all authors). All authors read and agreed on the final submitted version of the manuscript.

## Funding

This work was supported by the National Heart, Lung, and Blood Institute R01‐HL153430; Lilly Endowment Inc.

## Conflicts of Interest

The authors declare no conflicts of interest.

## Supporting information


**Appendix A:** Description of two‐compartment phantom studies.
**Figure S1:** In vivo data for the proposed hybrid 2D/3D sequence confirming that spins inside the excited 3D volume are at steady state before 2D imaging starts.
**Figure S2:** Simulation results that compare the effect of fast water exchange rate vs. in vivo water exchange rate on relative iMBV change.
**Figure S3:** Simulation results to investigate the impact of imperfect slice profile.
**Figure S4:** Summary of the repeatability experiments on three pigs by retrospectively truncating the k‐space acquisition into three acquisitions and measuring the ES‐to‐ED iMBV change.
**Figure S5:** Conceptual figure with typical MOLLI acquisition for myocardial T1 mapping (shown for the diastolic phase) and description of how spin history can confound accurate systolic vs. diastolic distinction.
**Figure S6:** In vivo experiment to support the feasibility of free‐breathing scans using the proposed method.

## Data Availability

Although the proposed method is currently an investigational technique and not yet tested in a clinical setting, we have openly shared the simulation codes for generating the results in Figure 4 and Suppl. Figure S3, as well as an example in vivo ferumoxytol‐enhanced myocardial MRI dataset (raw radial k‐space data) with the corresponding image reconstruction code to promote reproducible research. We have made these datasets and source codes available in the following GitHub page: https://github.com/TIM‐Lab/Cyclic‐iMBV.
