# Supplementary material for: Ferumoxytol‐Enhanced Myocardial T1 Tracking Using a Hybrid 2D/3D Steady‐State MRI Sequence Captures Cyclic Intramyocardial Blood Volume Dynamics
Source: NMR Biomed. 2026 May 18;39:e70308. doi: 10.1002/nbm.70308 (PMC13184369; doi:10.1002/nbm.70308)
Supplement: Supplementary file 1 — Appendix A: Description of two‐compartment phantom studies. Figure S1: In vivo data for the proposed hybrid 2D/3D sequence confirming that spins inside the excited 3D volume are at steady state before 2D imaging starts. Figure S2: Simulation results that compare the effect of fast water exchange rate vs. in vivo water exchange rate on relative iMBV change. Figure S3: Simulation results to investigate the impact of imperfect slice profile. Figure S4: Summary of the repeatability experiments on three pigs by retrospectively truncating the k‐space acquisition into three acquisitions and measuring the ES‐to‐ED iMBV change. Figure S5: Conceptual figure with typical MOLLI acquisition for myocardial T1 mapping (shown for the diastolic phase) and description of how spin history can confound accurate systolic vs. diastolic distinction. Figure S6: In vivo experiment to support the feasibility of free‐breathing scans using the proposed method. [file NBM-39-e70308-s001.docx]

**SUPPORTING INFORMATION** for *Unal et al. Ferumoxytol-enhanced myocardial T1 tracking with a hybrid 2D/3D spoiled steady-state sequence captures cyclic intramyocardial blood volume dynamics*

**LIST OF CONTENTS:**

- **Appendix A.** Description of two-compartment phantom studies.
- **Supplementary Figure S1:** In-vivo data for the proposed hybrid 2D/3D sequence confirming that spins inside the excited 3D volume are at steady state before 2D imaging starts.
- **Supplementary Figure S2:** Simulation results that compare the effect of fast water exchange rate vs. in vivo water exchange rate on relative iMBV change.
- **Supplementary Figure S3:** Simulation results to investigate the impact of imperfect slice profile.
- **Supplementary Figure S4:** Summary of the repeatability experiments on three pigs by retrospectively truncating the k-space acquisition into three acquisitions and measuring the ES-to-ED iMBV change.
- **Supplementary Figure S5:** Conceptual figure with typical MOLLI acquisition for myocardial T1 mapping (shown for the diastolic phase) and description of how spin history can confound accurate systolic vs. diastolic distinction.
- **Supplementary Figure S6:** In vivo experiment to support the feasibility of free-breathing scans using the proposed method.

**Appendix A. Description of two-compartment phantom studies:**

As shown in Fig 3a, we built two-compartment phantoms, attempting to realistically mimic the presence of blood-filled microvessels (“intravascular compartment”) inside myocardial tissue (“extravascular compartment”) using glass capillaries (“microvessels”), agar gel (“tissue”) and T1-shortening contrast agents (“blood-like solution”). As described in Fig. 3b, we designed two phantoms: one pre-contrast (pre-FE) phantom (top panel) and one post-contrast (post-FE) phantom, which differed only in terms of the amount of ferumoxytol used in the blood-like (intravascular) compartment. Furthermore, each of the two phantoms included two different fractional volumes (*f*_v,1_ and *f*_v,2_) as shown. We created a gel-like extravascular compartment for the two phantoms by doping agar (HiMedia Laboratories, Maharashtra, India) with gadolinium-based contrast agent (Gadavist, Bayer Healthcare, Whippany, NJ, USA). We formed cylindrical cavities in the agar gel by inserting 1.65-mm-diameter glass capillary tubes (Dagan Corporation, Minneapolis, MN, USA) in 3x3 and 4x4 grids to achieve two different fractional volumes. We then filled the intravascular compartment in each phantom by submerging the agar gel in the blood-like solutions for the pre-FE phantom (Fig. 3c, top) and post-FE phantom (Fig. 3c, bottom). We used a micro-wick (Hampton Research, Aliso Viejo, CA, USA) to remove any air bubbles formed inside the cylindrical cavities. Figure 3d shows an ultra-high-resolution 3D scan of the post-FE phantom (side-view on the top-left and top view on the top-right) which confirms the two-compartment structure of the phantom, i.e., cavities filled with post-FE blood-like solution. The bottom panel in Fig. 3d, which shows the zoomed-in view of the 2D cross-section, further verifies that the intravascular cross-sections are formed as intended. Next, we imaged the phantoms using the proposed hybrid 2D/3D SPGR sequence with multiple flip angles (FA = 10°, 15°, 20°, 25°, 36°). The upper range of the flip angle was dictated by the allowable SAR on the 3T clinical scanner. We normalized each T1-weighted acquisition (the five FAs ranging from 10° to 36°) to a PD-weighted scan to perform T1 mapping as described above. We set the in-plane resolution to 2.3x2.3 mm² to ensure sufficient partial volume effects between the intravascular (filled cavities) and extravascular (gadolinium-doped agar) compartments, simulating intramyocardial microvessels. Figure 3e verifies the realistic “mixing” of the two compartments in the T1 maps. To assess the impact of flip angle on the exchange-independence condition described in Eq. [3], we estimated fractional volumes using both the fast-exchange (T1-based in Eq. [1]) and the no-exchange (signal intensity-based in Eq. [2]) and formulas for each of the five FAs. These measurements were in turn used to determine the ground-truth fractional volumes based on the fact that the iMBV calculated using the closed-form expressions in Eq. [1] and Eq. [2] converge to the true iMBV as FA is increased per the work by Donahue et al^36^ as was described in the “Theory” section in Methods.

**Supplementary Figure S1: In-vivo data for the proposed hybrid 2D/3D sequence confirming that spins inside the excited 3D volume are at steady state before 2D imaging starts.**


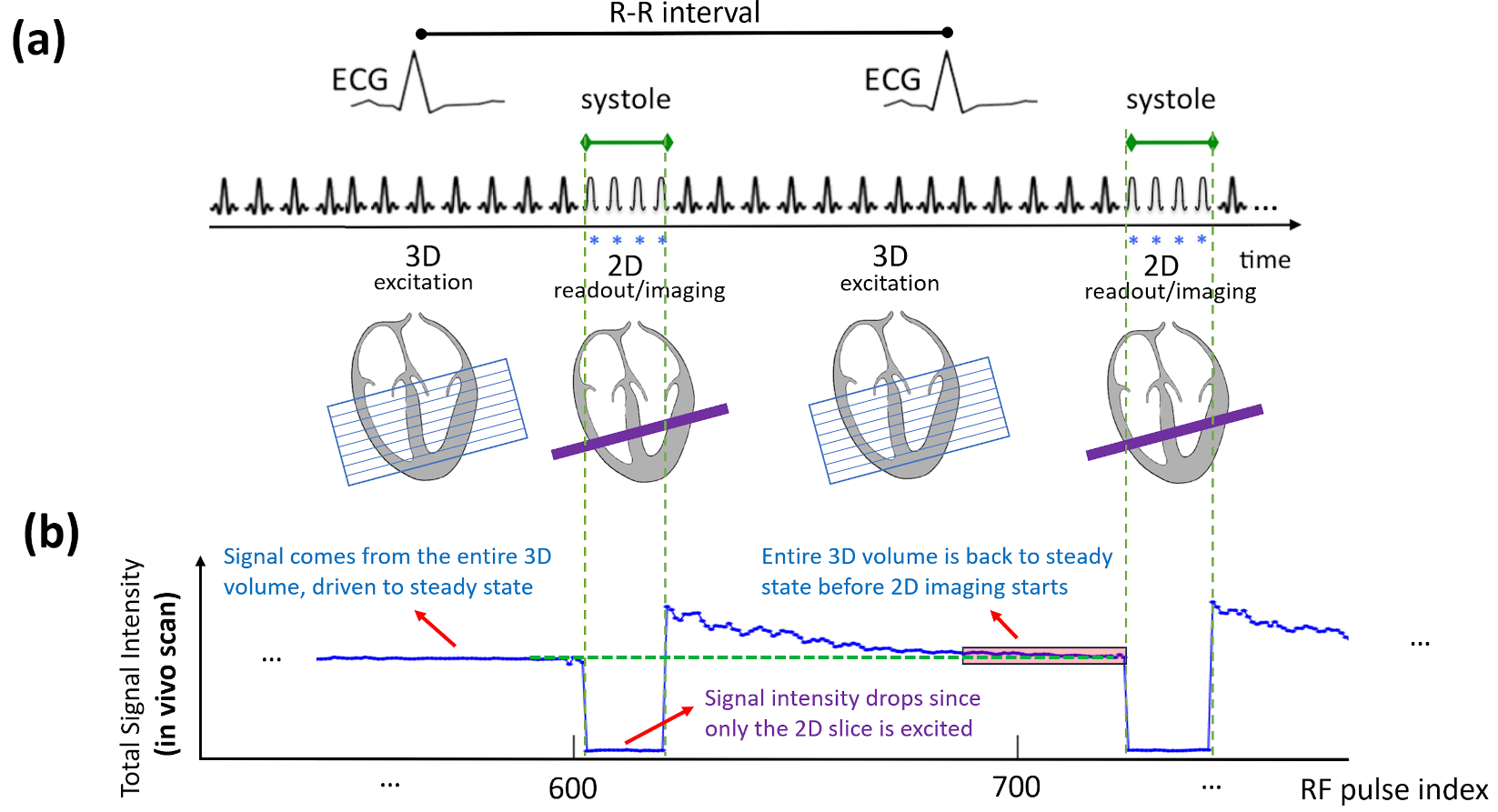


**Description of Supplementary Figure S1: (a)** The proposed hybrid 2D/3D T1-tracking pulse sequence wherein 3D and 2D RF pulses are applied continuously with golden-angle radial RF-spoiled gradient-recalled echo (SPGR) readouts. The 3D pulses excite the spins inside a large volume covering the heart (blue stack of slices), and the 2D pulses excite the spins inside the slice to be imaged. The diagram shown corresponds to end systolic acquisition. **(b)** Plot of the total signal intensity (blue dots) for one of the in vivo studies (integral of the 1D inverse Fourier Transform of each projection) as a function of time (RF pulse index). Note that, in the proposed pulse sequence shown in Fig. 2, we have continuous 2D/3D readouts, i.e., signal is acquired in both 3D slab excitations and 2D slice excitations. During 2D imaging, total signal intensity is much lower as expected (since there is a smaller number of excited spins in the 2D slice vs. the 3D slab). The key observation here is that, following the 2D readout/imaging period (systolic phase), the total signal intensity is not at steady-state initially since the out-of-slice spins have slowly recovered to equilibrium magnetization during the brief 2D imaging period ($\approx$90 ms). However, enabled by continuous 3D excitation pulses that follow the 2D imaging readout, the total signal intensity is driven back to steady state before 2D imaging starts in the next heartbeat, hence enabling steady-state 2D imaging that is robust to the confounding effects of out-of-slice magnetization (through-plane motion and in-flow effects).

**Supplementary Figure S2: Simulation results that compare the effect of fast water exchange rate vs. in vivo water exchange rate on relative iMBV change.**

**
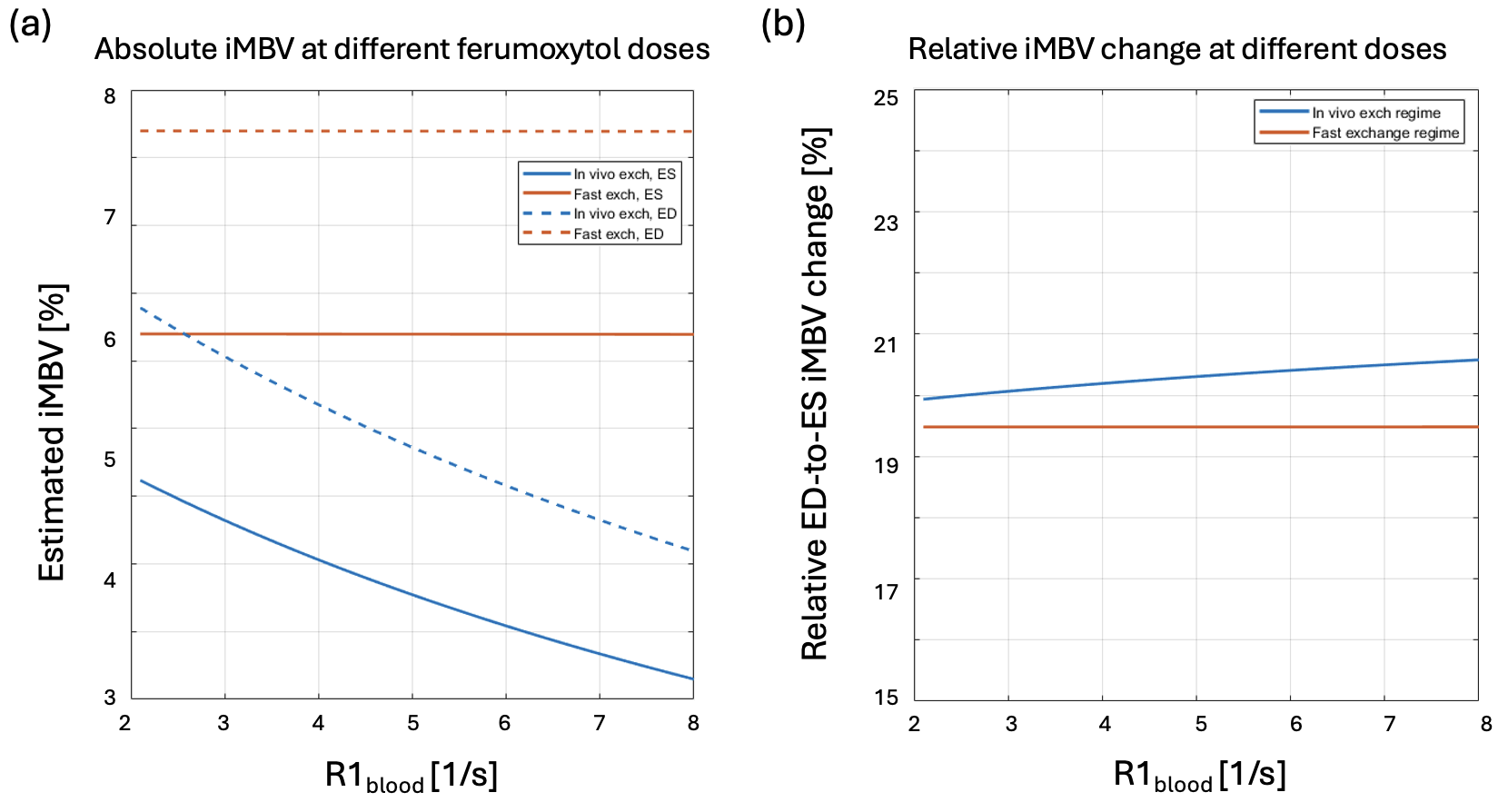
**

**Description of Supplementary Figure S2: (a)** We performed realistic simulations using the well-established two-compartment model using different exchange rates (ki = 11 Hz for in vivo exchange rate, and ki = 10 kHz for fast exchange rate). The other parameters were adopted from the results of our in vivo experiments (post-ferumoxytol blood R1 range, iMBV values during ES and ED, etc). iMBV values were estimated for different ferumoxytol doses (i.e., R1_blood_ = 2 to 8 s^-1^) using fast-exchange formula on both exchange conditions. As can be seen, the fast-exchange formula estimates ES iMBV (6.2%) and ED iMBV (7.7%) without any error under fast-exchange conditions (red curves), as expected since the exchange regime and the model are consistent. In the case of in-vivo exchange rate, however, the fast-exchange assumption leads to more and more error (up to 40-50%) in the iMBV values as the ferumoxytol dose increases (blue curves). **(b)** On the other hand, the relative iMBV change from ED to ES under the in-vivo exchange regime (blue curve) remains within 1% absolute difference (i.e., approximately 5% relative difference) compared to fast-exchange regime (red curve) in the same ferumoxytol-dose range. This feature can be attributed to the fact that the inconsistency between the water exchange regime and the model estimating iMBV causes similar extent of underestimation, most of which cancels out when relative iMBV change is measured.

**Supplementary Figure S3: Simulation results to investigate the impact of imperfect slice profile.**


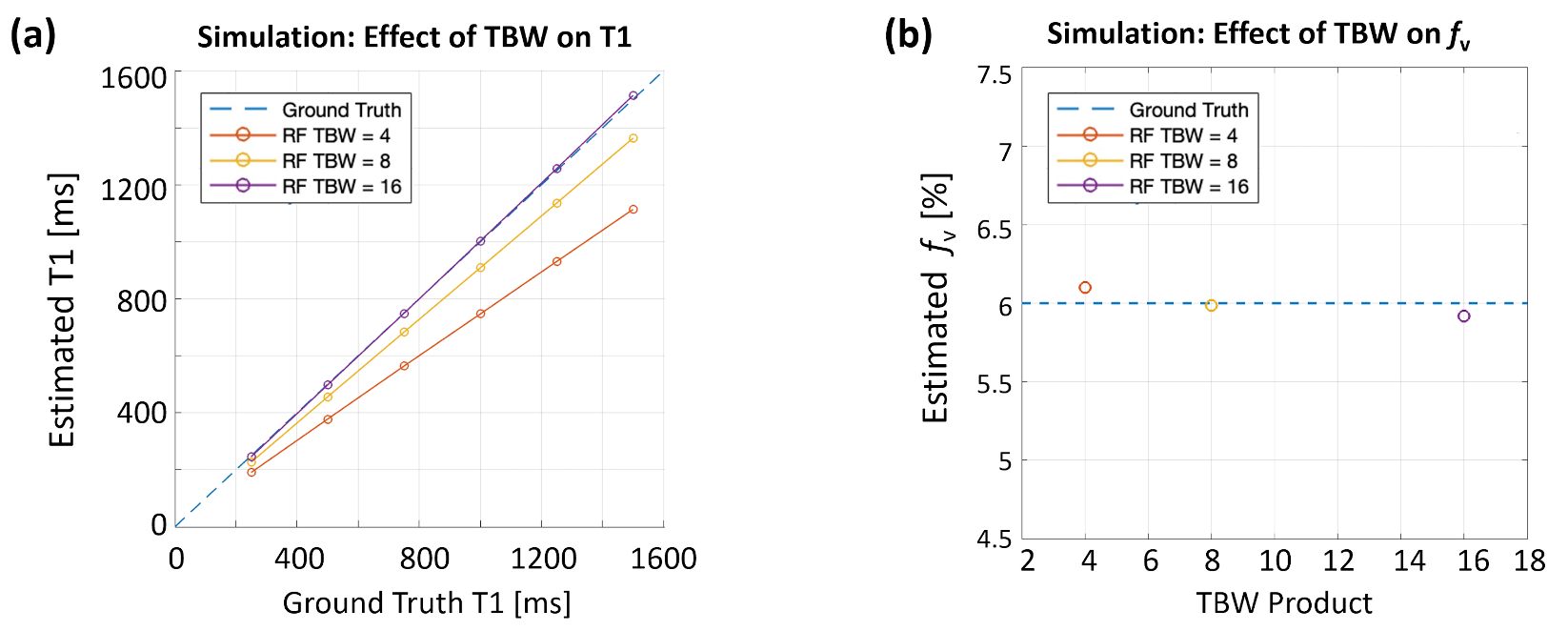


**Description of Supplementary Figure S3: (a)** We performed realistic Bloch equation simulations to investigate how imperfections in the slice profile due to limited time bandwidth product (TBW) affect **(a)** estimated T1 and **(b)** fractional volume (*f_v_*). **(a)** As expected, lower TBW (i.e., more imperfect slice profile) results in larger errors in the estimated T1 (up to 20% for low TBW product). **(b)** Despite large errors in estimated T1, the resulting estimated fractional volume (*f_v_*) is quite insensitive (within 2% relative error) for all TBWs. This is because the error in estimated T1 (pre-contrast and post-contrast) is partially cancelled out when fractional volume (simulated iMBV) is calculated per Eq. [1].

**Supplementary Figure S4:** Summary of the repeatability experiments on three pigs by retrospectively truncating the k-space acquisition into three acquisitions and measuring the ES-to-ED iMBV change.


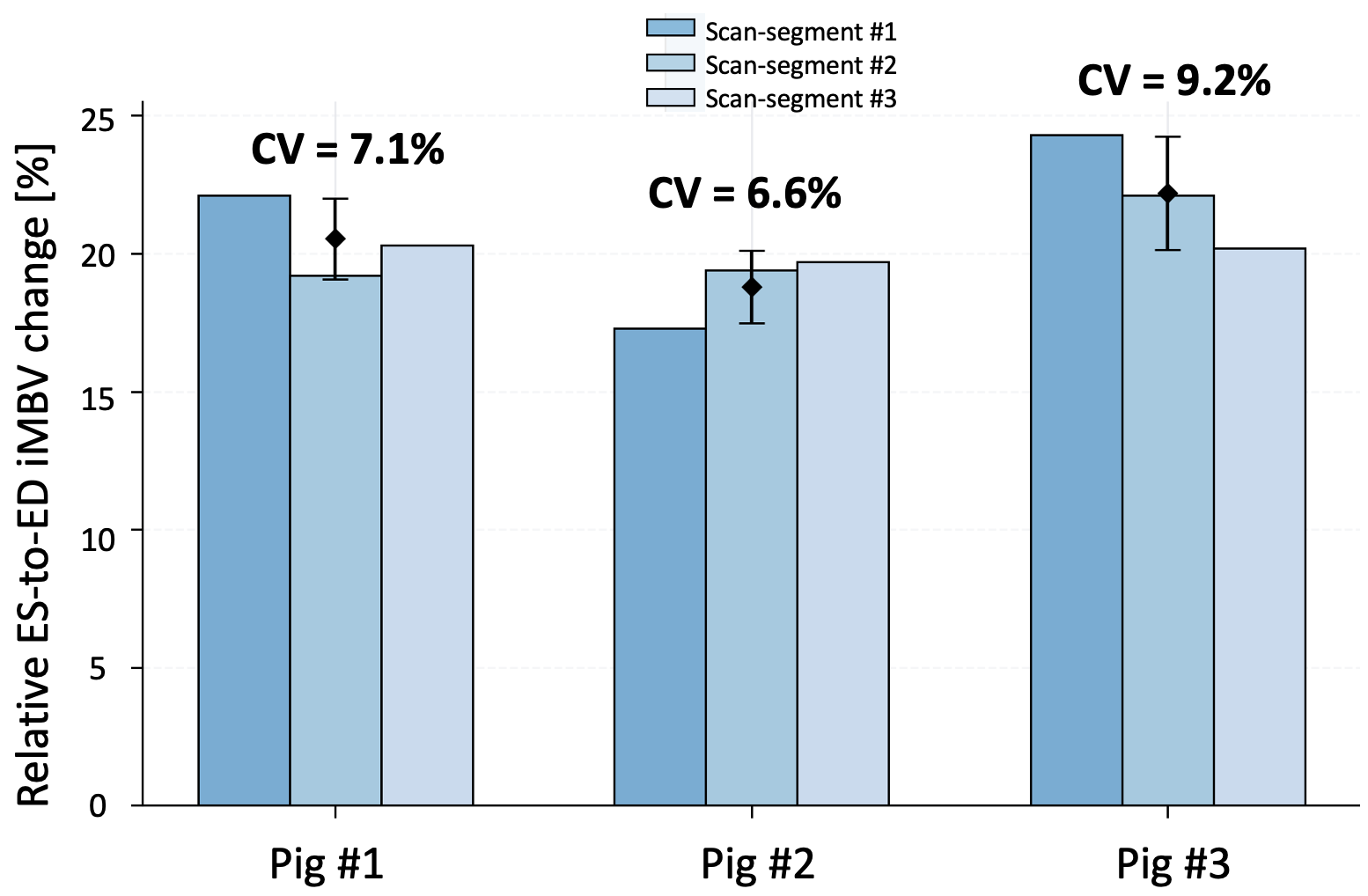


**Description of Supplementary Figure S4:** We retrospectively evaluated the relative ES-to-ED iMBV differences between 3 sets of measurements in a subset of the animals. The resulting ES-to-ED iMBV values and the corresponding coefficient of variation (CV) was within the range reported in the literature: 20.53% ± 1.45% for the first study (CV = 7.1%), 18.82% ± 1.24% for the second study (CV = 6.6%), 22.20% ± 2.05% for the third study (CV = 9.2%). It is worth noting that the primary factor contributing to the CV for each animal study is the reduced SNR due to retrospective truncation of acquired data (67% reduction in the number of k-space projections) which reduces the SNR. This can potentially be overcome in future work by using deep-learning-based denoising algorithms or model-based iterative image reconstruction to improve SNR.

**Supplementary Figure S5: Conceptual figure with typical MOLLI acquisition for myocardial T1 mapping (shown for the diastolic phase) and description of how spin history can confound accurate systolic vs. diastolic distinction.**


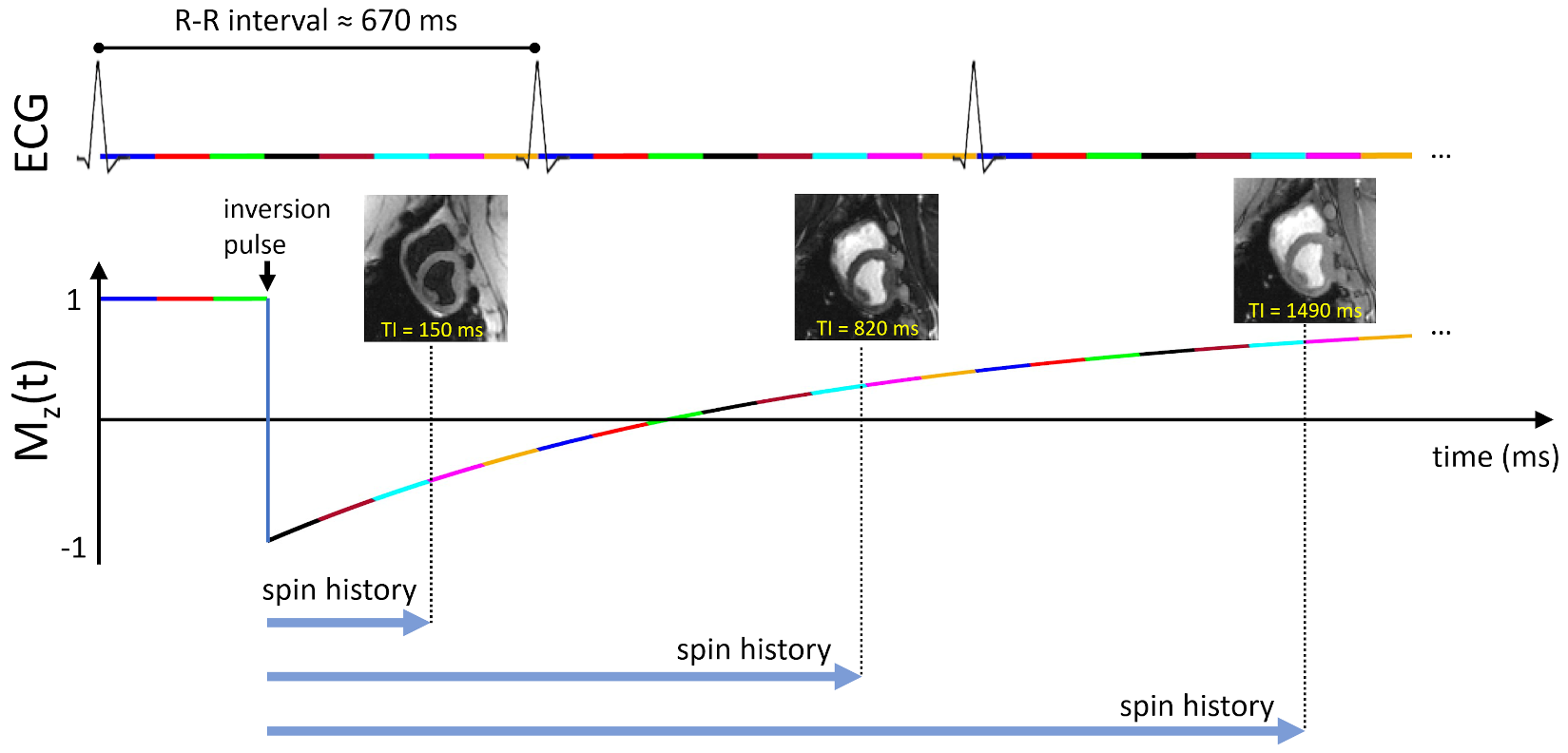


**Description of Supplementary Figure S5:** In a typical MOLLI scheme, an inversion pulse is applied, synchronized with the R-wave, and multiple images with different inversion times are acquired to perform T1 fitting with Look-Locker correction. However, images with inversion times longer than the R-R interval reflect the spin history, i.e., recovery of the spin state after the inversion pulse, from not only the current cardiac phase, but also the preceding cardiac phases including the previous R-R interval. For instance, in this concept figure, the inversion recovery curve corresponding to the image with inversion time of “TI = 820 ms” passes through a series of cardiac phases (shown with different colors in the ECG diagram as well as the T1 recovery curve) which reflects the fact that the “spin history” of this image (highlighted by the corresponding blue arrow at the bottom) spans the time period from the inversion pulse to the 2D readout time point which is the diastolic phase of the 2^nd^ heartbeat. This mixture of systolic/diastolic spin history in MOLLI can make it challenging or infeasible to detect systolic vs. diastolic differences reliably.

**Supplementary Figure S6: In vivo experiment to support the feasibility of free-breathing scans using the proposed method.**

**
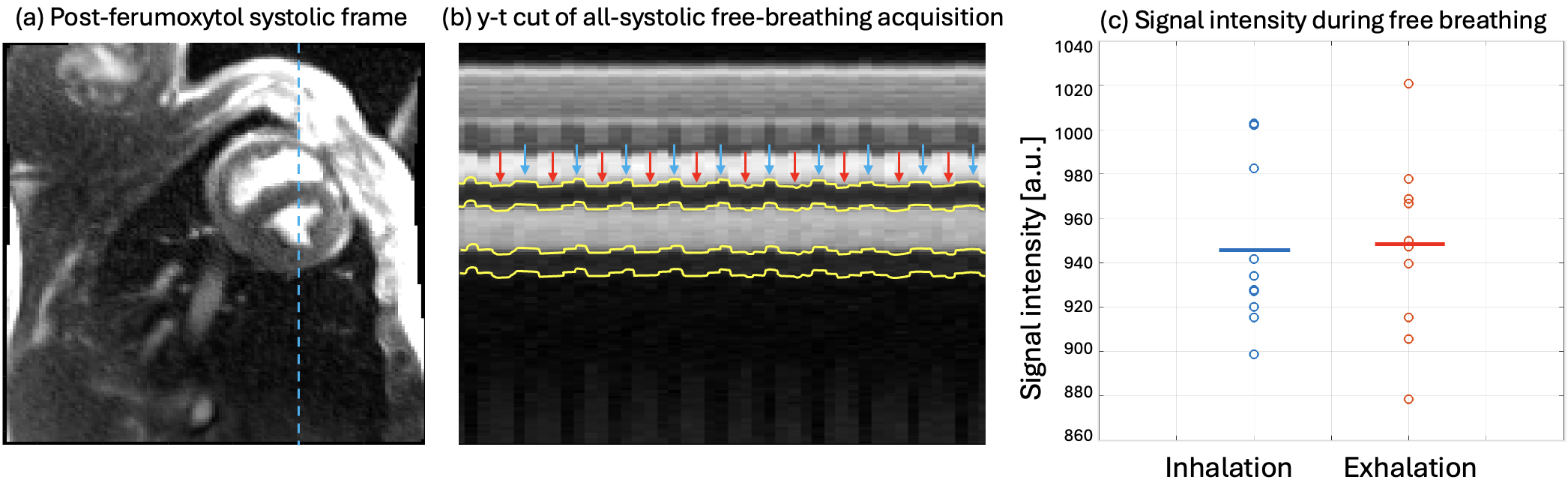
**

**Description of Supplementary Figure S6: (a)** An example systolic frame from a series of systolic images acquired after ferumoxytol infusion under free-breathing conditions over 10 respiratory cycles. **(b)** The “y-t cut” across the blue dashed line in panel a demonstrates the breathing motion with inhalation and exhalation phases highlighted using blue and red arrows. The yellow lines show the segmentation of septal myocardium and the inferno-lateral wall, highlighting the breathing pattern. (c) Each dot represents averaged septal pixel intensities in one cardiac cycle (blue for inhalation, red for exhalation) for 10 respiratory cycles. **(c)** As can be seen in the figure, the difference in averaged signal intensities across 10 respiratory cycles is less than 0.5% which is within the noise level (mean signal intensity = 945 for inhalation, mean signal intensity = 947 for exhalation). This feasibility experiments suggests that the proposed hybrid 3D excitation scheme maintains T1-weighted steady state is maintained under free-breathing conditions.
